# PHB2 promotes tumorigenesis *via* RACK1 in non-small cell lung cancer

**DOI:** 10.7150/thno.52848

**Published:** 2021-01-01

**Authors:** Bin Wu, Ning Chang, Hangtian Xi, Jie Xiong, Ying Zhou, Yingtong Wu, Shuo Wu, Ning Wang, Hongyu Yi, Yun Song, Lihua Chen, Jian Zhang

**Affiliations:** 1 Department of Respiratory and Critical Care Medicine, Xijing Hospital, Fourth Military Medical University, Xi'an, Shaanxi 710032, China;; 2 Department of Immunology, Fourth Military Medical University, Xi'an, Shaanxi 710032, China;; 3 Department of Infectious Diseases, Tangdu Hospital, Fourth Military Medical University, Xi'an, Shaanxi 710032, China

**Keywords:** prohibitin 2, receptor for activated C kinase 1, non-small cell lung cancer, tumorigenesis, integrin β1

## Abstract

**Background:** Lung cancer has the highest mortality rate among cancers worldwide, with non-small cell lung cancer (NSCLC) the most common type. Increasing evidence shows that PHB2 is highly expressed in other cancer types; however, the effects of PHB2 in NSCLC are currently poorly understood.

**Method:** PHB2 expression and its clinical relevance in NSCLC tumor tissues were analyzed using a tissue microarray. The biological role of PHB2 in NSCLC was investigated in vitro and in vivo using immunohistochemistry and immunofluorescence staining, gene expression knockdown and overexpression, cell proliferation assay, flow cytometry, terminal deoxynucleotidyl transferase dUTP nick end labeling (TUNEL) assay, wound healing assay, Transwell assay, western blot analysis, qRT-PCR, coimmunoprecipitation, and mass spectrometry analysis.

**Results:** Our major finding is that PHB2 facilitates tumorigenesis in NSCLC by interacting with and stabilizing RACK1, which further induces activation of downstream tumor-promoting effectors. PHB2 was found to be overexpressed in NSCLC tumor tissues, and its expression was correlated with clinicopathological features. Furthermore, PHB2 overexpression promoted proliferation, migration, and invasion, whereas PHB2 knockdown enhanced apoptosis in NSCLC cells. The stimulating effect of PHB2 on tumorigenesis was also verified in vivo. In addition, PHB2 interacted with RACK1 and increased its expression through posttranslational modification, which further induced activation of the Akt and FAK pathways.

**Conclusions:** Our results reveal the effects of PHB2 on tumorigenesis and its regulation of RACK1 and RACK1-associated proteins and downstream signaling in NSCLC. We believe that the crosstalk between PHB2 and RACK1 provides us with a great opportunity to design and develop novel therapeutic strategies for NSCLC.

## Introduction

Lung cancer is an increasingly global problem and public health issue 1. One-quarter of all cancer-related deaths worldwide are due to lung cancer, although extensive efforts have been made to improve related diagnostic technologies and medical treatments in recent years 2. Every year, 1.8 million people are diagnosed with lung cancer, and 1.6 million people die to this disease 3. Lung cancer can be divided into small cell lung cancer (SCLC) and non-small cell lung cancer (NSCLC), with NSCLC accounting for >85% of all cases 4. Over the past two decades, select patients with NSCLC have benefited from small molecule tyrosine kinase inhibitor application and immunotherapy, achieving unprecedented survival rates 5. However, the overall survival and cure rates for NSCLC remain low, particularly in advanced cases 5, 6. In addition, studies of crucial factors contributing to tumorigenesis in NSCLC are still limited. Therefore, studies identifying new molecular targets and exploring novel mechanisms for this fatal malignancy are urgently needed.

Prohibitin 2 (PHB2), also known as BAP37, REA, or prohibitone, is a highly conserved ubiquitous eukaryotic protein. It belongs to the prohibitin (PHB) family and is encoded by the nuclear gene *PHB2* located on chromosome 12p13 7. PHB2 is a multifunctional protein involved in numerous vital cellular processes including signal transduction, gene transcription, cell survival, metabolism, inflamemation, and apoptosis 8, 9. Increasing evidence shows that PHB2 is highly expressed in prostate cancer 10, liver cancer 11, esophageal squamous cell carcinoma 12, and diffuse large B cell lymphomas 13 compared with normal tissues. Moreover, a correlation between PHB2 expression and patients' clinicopathologic characteristics has been observed, and PHB2 is regarded as an independent prognostic marker for these cancers 10-13. Furthermore, PHB2 depletion has been shown to inhibit cancer cell growth and metastasis and enhance apoptosis in vitro and in vivo 10-14. However, the role of PHB2 in NSCLC is currently poorly understood.

Receptor for activated C kinase 1 (RACK1), a member of the tryptophan-aspartate repeat (WD-repeat) family of proteins, is a highly conserved intracellular adaptor protein with significant homology to the β subunit of G-proteins (Gβ) 15. RACK1 has seven β-propeller blades that serve as binding sites for multiple interaction partners, and it acts as a scaffolding protein, making it a key mediator of various pathways that contribute to numerous aspects of cellular function 16.

Numerous studies indicate that RACK1 plays an important role in cancer progression and that its expression is altered during angiogenesis and in many human carcinomas 15, 16. The function of RACK1 in tumorigenesis is cancer-type specific. RACK1 was found to be downregulated and act as a tumor suppressor in gastric cancer 17. In contrast, RACK1 was found to be upregulated and contribute to tumorigenesis in breast cancer 18, hepatocellular carcinoma 19, melanoma 20, pulmonary adenocarcinoma 21, 22, pancreatic ductal adenocarcinoma 23, and neuroblastoma 24. Thus, RACK1 is considered to be a crucial factor affecting the development and progression of cancer. However, the mechanism and role of RACK1 and RACK1-associated proteins in NSCLC have not yet been fully elucidated.

In the present study, we sought to explore the potential function of PHB2 in NSCLC. Our results demonstrate for the first time that PHB2 is upregulated in human NSCLC compared to normal controls and that elevated expression of PHB2 is associated with the clinicopathological parameters of patients with NSCLC. Mechanistically, knockdown or overexpression of PHB2 leads to suppression or enhancement of NSCLC cell phenotypes, respectively. In addition, PHB2 promotes tumorigenesis in NSCLC by interacting with and stabilizing RACK1, further inducing changes to downstream effectors. These findings suggest that PHB2 acts as an oncogene in NSCLC and may be used as a new therapeutic target for NSCLC.

## Materials and Methods

### Cells and cell culture

Human NSCLC cell lines (A549, H1299, PC-9, HCC827, and H1975) and the normal human lung epithelial cell line BEAS-2B were purchased from the Shanghai Cell Bank of the Chinese Academy of Sciences. All NSCLC cell lines were seeded in culture flasks and cultured in RPMI 1640 medium (Gibco, Thermo Fisher Scientific, Inc.) supplemented with 10% fetal bovine serum (Gibco) and 1% penicillin-streptomycin (Gibco) at 37 °C in the presence of 95% O_2_ and 5% CO_2_. BEAS-2B cells were cultured in Dulbecco's modified Eagle's minimal essential medium (DMEM, Gibco) under consistent culture conditions.

### NSCLC tissue samples

A human NSCLC tissue microarray containing 48 patient samples was purchased from Xi'an Alenabio.com (Shaanxi, China). Within the cohort, there were 48 paired cancer and adjacent normal pulmonary specimens. All patients had detailed clinical parameters. The clinical features of the patients are shown in Table S1. There were a total of 23 women and 25 men in this study, with an age range between 32 and 75 years old. The study protocol was approved by the Fourth Military Medical University Ethics Committee.

### Immunohistochemistry

Immunohistochemistry (IHC) staining was performed according to standard protocols. Briefly, tissue microarrays were deparaffinized in 100% xylene and rehydrated in a descending ethanol series. Tissue microarrays were neutralized with 3% H_2_O_2_ for 15 min and then incubated with goat serum for 60 min at room temperature. After antigen retrieval, the tissue microarrays were incubated with primary antibody in a humidified box overnight at 4 °C. The tissue microarrays were then washed twice with PBS and incubated with horseradish peroxidase (HRP)-conjugated secondary antibody for 40 min at room temperature. Then, the tissue microarrays were stained with 3,3'-diaminobenzidine (DAB), counterstained with hematoxylin, mounted in neutral gum, and analyzed using a brightfield microscope.

IHC staining was scored and classified based on the following criteria, as described previously 25: (a) the proportion of positive cells (0, ≤5%; 1, 6-25%; 2, 26-50%; 3, 51-75%; 4, >75%) and (b) the staining intensity (0, negative; 1, week; 2, moderate; 3, strong). The scores were multiplied to obtain the total score, and the average score was employed to distinguish low and high PHB2 or RACK1 expression in NSCLC samples. All sections were scored and calculated by two independent pathologists.

### Cell proliferation assay

Cell Counting Kit-8 (CCK-8, Beyotime, Shanghai, China) assay was used to evaluate the function of PHB2 on cell proliferation following the manufacturer's instructions. Cells in the logarithmic growth phase were seeded in 96-well culture plates and cultured under normal conditions for 5 days. The medium was replaced with fresh basal medium containing 10% (v/v) CCK-8 reagent for 1 h at 37 °C. The optical density (OD) was recorded at 450 nm with a microplate reader (Rayto, Ltd., China).

### Colony forming assay

Approximately two hundred cells with stable knockdown or overexpression of PHB2 were seeded in 6-well plates in triplicate. Cells in each group were cultured for 14 days at 37 °C in the presence of 95% O_2_ and 5% CO_2_. The cells were fixed with methanol for 10 min and stained with 0.1% crystal violet solution. The number of colonies was counted and imaged.

### Flow cytometric cell cycle and cell apoptosis analysis

The cell cycle distribution was measured with a cell cycle assay kit (Beyotime) according to the manufacturer's protocols. Briefly, cells with stable PHB2 knockdown were harvested and fixed in ice-cold 70% ethanol overnight. After washing twice with ice-cold PBS, the cells were resuspended in PBS (500 μL) and stained with 50 μg/mL propidium iodide (PI) solution and 100 μg/mL RNase A, followed by cell cycle analysis with the MoFlo XDP Flow Cytometry System (Beckman Coulter, CA, USA). Annexin V and PI double staining kits (BioLegend) were used to evaluate the role of PHB2 in apoptosis according to the manufacturer's instructions. Briefly, cells were seeded in 6-well plates at a density of 2 × 10^5^ cells/well and cultured normally for 48 h. Then, the cells were resuspended in annexin V-binding buffer and stained with annexin V-phycoerythrin (PE) and PI (1 µg/mL). After incubation at room temperature, apoptotic cells were quantified by flow cytometry (Beckman).

### TUNEL assay

A TUNEL assay kit (In Situ Cell Death Detection Kit; Roche Diagnostics) was also used to evaluate the apoptosis ratio of NSCLC cancer cells, as described previously 26. Briefly, fluorescein-dUTP was employed to identify apoptotic cell nuclei, and 4',6-diamidino-2-phenylindole (DAPI) (Sigma-Aldrich) was used to stain all cell nuclei. The apoptotic index was calculated as the ratio of TUNEL-positive cells to the total number of DAPI-positive cells within the same area from five randomly selected fields in each group.

### Immunofluorescence analysis

Cells were fixed with 4% paraformaldehyde for 15 min at 37 °C. The fixed cells were then permeabilized with 0.2% Triton X-100 (Sigma-Aldrich, T8787) for 15 min at room temperature, blocked, incubated with primary antibodies overnight at 4 °C, and then incubated with secondary antibodies at room temperature for 1 h. After washing, DAPI was used to stain the cell nuclei. Colocalization of PHB2 and RACK1 was detected with an Olympus FV1000 confocal laser scanning microscope.

### Wound healing assay

A wound healing assay was performed to determine the migratory ability of the cells. Transfected A549 and H1299 cells were seeded in 6-well plates and grown to confluence. Two hundred-microliter pipette tips were used to scrape the confluent cell monolayers, and cell debris was washed off twice with PBS. The gaps between wound edges were imaged at time points 0 h and 48 h after wounding. The wound healing rate (%) was evaluated by comparing differences in wound width.

### Transwell assay

Invasion assays were carried out using 24-well BioCoat Matrigel Invasion Chambers (BD, Biosciences, Franklin Lakes, USA) according to the manufacturer's protocol. Cells cultured in serum-free media for 12 h were resuspended, and 2 × 10^4^ cells were seeded into the upper Transwell chamber. Cells were cultured with serum-free medium in the upper chamber, and the lower chamber contained 600 μL medium with 10% FBS. The cells were incubated at 37 °C and 5% CO_2_ for 24 h. The cells on the upper surface of the filters were gently wiped with a cotton swab. Invaded cells on the lower surface were fixed with 10% methanol for 5 min and stained with 0.1% crystal violet (Sigma-Aldrich) for 10 min. The invaded cells were then imaged and counted under a microscope. For the migration assays, the procedures were performed as for the invasion assays but the filters were not precoated with Matrigel.

### RNA extraction and quantitative real-time PCR

Adherent cells with stable knockdown or overexpression of PHB2 were cultured for 48 h. TRIzol reagent (Invitrogen, Carlsbad, CA, USA) was used to extract total RNA from treated cells according to the manufacturer's instructions. cDNA was prepared and obtained using the PrimeScript RT Master Mix Perfect Real Time Kit (TaKaRa, Otsu, Shiga, Japan). Using cDNA as the template, a quantitative real-time PCR assay was performed with SYBR Premix ExTaq (TaKaRa) on a CFX96 real-time PCR detection system (Bio-Rad, Hercules, CA, USA). The reaction conditions were defined as follows: predenaturation step at 95 °C for 10 s, 40 cycles at 95 °C for 15 s, and 60 °C for 60 s. The β-actin gene was employed as the reference gene. The primer sequences for qPCR were as follows: PHB2 forward: 5'-CCAAACAAGTGGCACTGAGC-3' and reverse: 5'-ACGATTCTGTGATGTGGCGA-3'; RACK1 forward: 5'-AGATAAGACCATCATCAT-3' and reverse: 5'-AGATAACCACATCACTAA-3'; and β-actin forward: 5'-TTGCCGACAGGATGCAGAAG-3' and reverse: 5'-AGGTGGACAGCGAGGCCAGGAT-3'.

### Western blot analysis

Western blot analysis was performed as previously described 26. Cells were harvested and homogenized with RIPA buffer containing protease inhibitor cocktail (Roche Diagnostics) on ice. After centrifugation at 12,000 ×*g* for 15 min at 4 °C, the total protein content of the supernatant was quantified using a bicinchoninic acid protein assay (Applygen Technologies, Inc.) and the supernatant was stored at -80 °C until use. Protein samples were separated by electrophoresis on 10% SDS-PAGE gels and transferred to polyvinylidene difluoride membranes (EMD Millipore). After blocking with 5% milk, the membranes were incubated with the primary antibodies overnight at 4 °C. The blots were then incubated with horseradish peroxidase-conjugated anti-rabbit or anti-mouse immunoglobulin (IgG) secondary antibody for 1 h at 37 °C. Immunoreactive bands were revealed with the ECL^TM^ Advance Western Blotting Detection Kit (Amersham Bioscience) and scanned with the ChemiDoc XRS system (Bio‑Rad Laboratories, Inc.). ImageJ version 1.8.0 software (National Institutes of Health) was employed to measure protein band intensity.

### Coimmunoprecipitation analysis

Coimmunoprecipitation (Co-IP) analysis was performed as previously described 27. Briefly, cells were washed with ice-cold PBS and lysed in buffer containing 20 mM Tris-HCl pH 7.5, 150 mM NaCl, 1% Triton X-100, and protease inhibitor on ice. The cell lysates were centrifuged at 12,000 ×*g* for 10 min at 4 °C. After preclearing the lysates with Protein A/G agarose beads (Santa Cruz) for 30 min at 4 °C, the supernatants were incubated with the indicated antibodies or control IgG antibody overnight at 4 °C to enrich the antigen-antibody complex. After mixing the lysate with Protein A/G agarose beads for 2 h at 4 °C, the immunocomplex was washed twice with PBS, resuspended and boiled with 1× SDS buffer at 95 °C for 10 min, and then subjected to immunoblotting analysis. Anti-IgG was used as a negative control.

### Antibodies

Antibodies against the following proteins were used: RACK1 (1:1000, Cell Signaling, Danvers, MA, USA), phospho-Akt (Ser473) (p-Akt, 1:1000, Cell Signaling), Akt (1:1000, Cell Signaling), phospho-FAK (Tyr397) (p-FAK, 1:1000, Cell Signaling), FAK (1:1000, Cell Signaling), HA (1:1000, Cell Signaling), PHB2 (1:500, Santa Cruz, CA, USA), integrin β1 (1:500, Santa Cruz), β-actin (1:1000, Santa Cruz) and β-tubulin (1:500, Proteintech, Wuhan, China). Horseradish peroxidase-conjugated secondary antibodies (anti-mouse/rabbit IgG) (1:5000, Cell Signaling) were used.

### In vivo tumor xenograft models

BALB/c nude mice (6-week-old males, 18-20 g) were purchased from the Laboratory Animal Center of the Fourth Military Medical University. All animal protocols were approved by the Fourth Military Medical University Ethics Committee on Animal Care, and all experiments were performed in adherence with the National Institutes of Health Guidelines on the Care and Use of Laboratory Animals. Mice were randomly divided into the indicated groups. To generate the tumor xenograft models, 7 × 10^6^ A549 cells with stable knockdown of PHB2 were resuspended in 50 µL PBS and subcutaneously injected into the flanks of nude mice. The tumor size of each nude mouse was measured every week for a total of 28 days. At the end of the experiment, the mice were sacrificed and the transplanted tumors were removed, weighed, and fixed for further study.

### Lentiviral preparation and infection

For stable transfection of short hairpin RNA (shRNA), predesigned shRNA-expressing lentivirus particles were synthesized (GeneChem, Shanghai, China) using the following shRNAs: shPHB2 #1 forward: 5′- CCAGAATATCTCCAAGACGAT-3′ and reverse: 5′-ATCGTCTTGGAGATATTCTGG-3′; shPHB2 #2 forward: 5′-GCTGAGCTTTAGCCGAGAGTA-3′ and reverse: 5′-TACTCTCGGCTAAAGCTCAGC-3′; and shControl (shCtrl), forward: 5′-TTCTCCGAACGTGTCACGT-3′ and reverse: 5′-ACGTGACACGTTCGGAGAA-3′. For overexpression of PHB2, a full-length cDNA encoding PHB2 whose C-terminus was fused with a cDNA fragment encoding flag was inserted into the pcDNA3.1 vector (Hanheng Biotechnology, Shanghai, China). Lentiviral infection of A549 and H1299 cells was performed according to the manufacturer's protocols. Briefly, cells were infected with the lentivirus for 48 h and selected with puromycin by increasing the concentration from 1 to 3 ng/mL for 1 week. The surviving cells were cultured for further research.

### siRNA transfection

RACK1-specific small interfering RNA (siRNA; Hanheng Biotechnology, Shanghai, China) was transfected using Lipofectamine 2000 reagent (Invitrogen, CA, USA) according to the manufacturer's protocol. The sequences of RACK1 siRNA were as follows: forward: 5′-CUCUGGAUCUCGAGAUAAAdTdT-3′ and reverse: 5′-UUUAUCUCGAGAUCCAGAGdTdT-3′. A nonspecific control siRNA (siControl) was used as a scrambled siRNA control. The sequences of the control siRNA were as follows: forward: 5′-UUCUCCGAACGUGUCACGUdTdT-3′ and reverse: 5′-ACGUGACACGUUCGGAGAAdTdT-3′.

### Ubiquitination assay

A549 cells with or without PHB2 overexpression were transfected with HA-Ub using Lipofectamine 2000 reagent (Invitrogen) and the cells were then treated with MG132 (10 μΜ) for 8 h before collection. The whole-cell lysates were subjected to immunoprecipitation with RACK1 antibody and western blotting with anti-HA antibody to detect ubiquitinated RACK1.

### Mass spectrometry analysis

Liquid chromatography and tandem mass spectrometry (LC-MS/MS) were performed as previously described 28. The general procedure is described in detail in Supplementary Materials.

### Statistical analysis

All data are expressed as mean ± SEM. The associations between PHB2 expression and clinicopathological parameters were analyzed by chi-square test or Fisher's exact test. The correlation between PHB2 expression and RACK1 expression was determined by Pearson's correlation test. Statistical comparisons between two groups were examined using paired *t*-test or unpaired *t-*test. Statistical comparisons among more than two groups were evaluated using one-way ANOVA followed by Student-Newman-Keuls test. The level of significance was set at *P* < 0.05*.* SPSS software package version 14.0 (SPSS, Chicago, IL, USA) was used for data analysis.

## Results

### PHB2 is overexpressed in NSCLC tumor tissues compared to normal controls, and its expression is correlated with clinicopathological features

**Figure 1:**
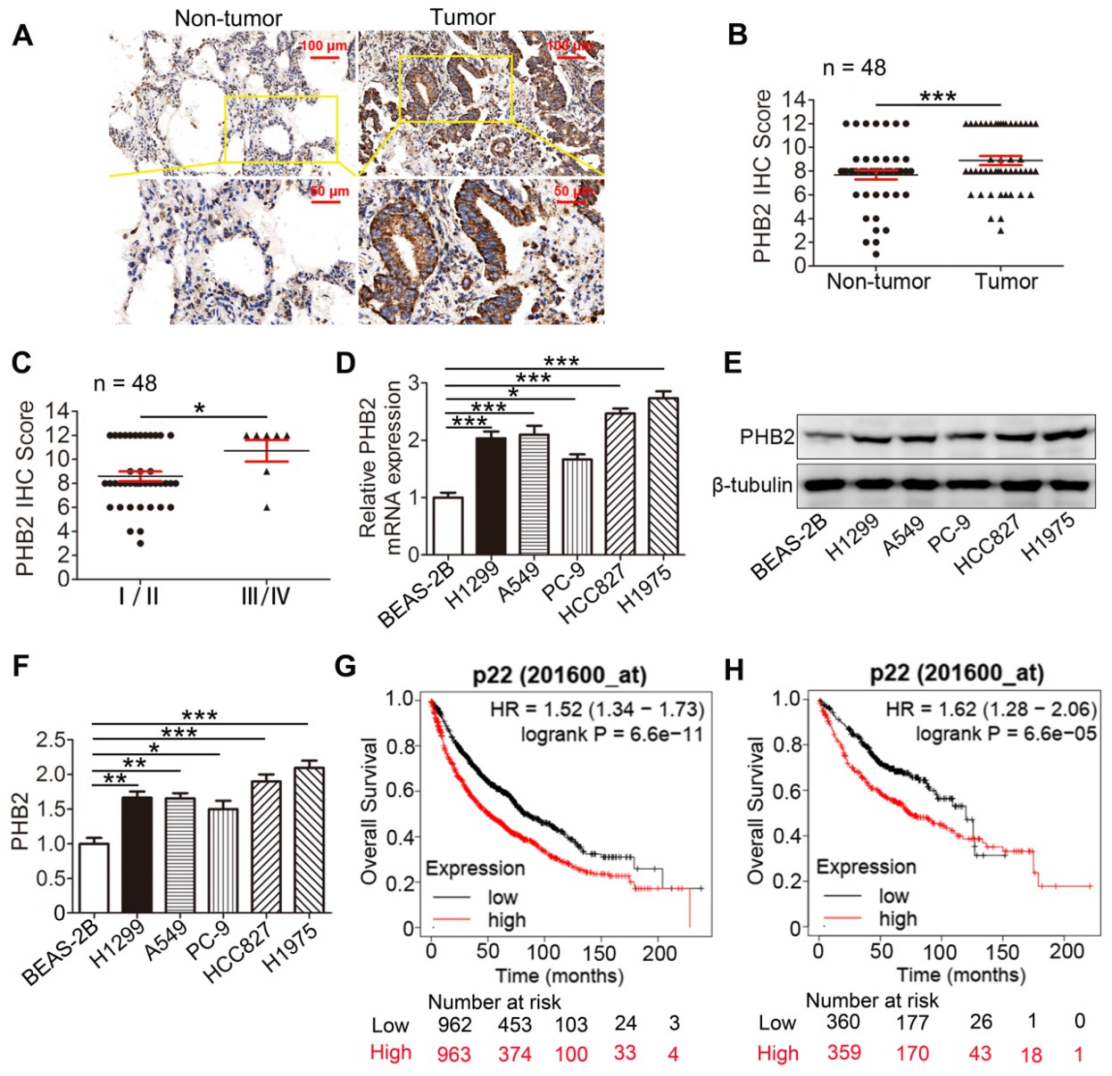
** PHB2 expression in NSCLC tissues and cell lines.** (A) Representative images of IHC staining for PHB2 in 48 paired NSCLC tumor tissues and adjacent normal tissues. (B) Quantitative analysis of PHB2 protein expression in tumor and tumor-adjacent tissues. (C) Quantitative analysis of PHB2 protein expression in NSCLC at advanced (stages III/IV) and early clinical stages (stages I/II). (D) mRNA level of PHB2 in five NSCLC cell lines (A549, H1299, PC-9, HCC827, and H1975) and the normal human bronchoalveolar epithelial cell line BEAS-2B. (E)-(F) Representative immunoblots and densitometric quantification for PHB2 and β-tubulin in five human NSCLC cell lines and BEAS-2B. (G)-(H) High expression of PHB2 was correlated with poor OS in patients with lung cancer (G) and lung adenocarcinoma (H). Data are presented as mean ± SEM. *P < 0.05; **P < 0.01; ***P < 0.001. Abbreviations: IHC, immunohistochemistry; OS, overall survival.

To evaluate the clinical significance of PHB2, PHB2 expression in 48 pairs of human clinical NSCLC tissues and their corresponding noncancerous lung tissues was examined by IHC (Table S1). The results indicate that PHB2 was mainly diffused throughout the cytoplasm and membrane of the cancer cells (Figure 1A). PHB2 expression in NSCLC was significantly higher than that in adjacent noncancerous lung tissues (Figure 1A-B). Moreover, higher PHB2 expression was observed in NSCLC patients with advanced clinical stage (stages III/IV) than in those with early clinical stage (stages I/II) (Figure 1C). We further detected expression of PHB2 in NSCLC cell lines including A549, H1299, H1975, HCC827, and PC-9 and the normal human bronchial epithelial cell line BEAS-2B by qRT-PCR and western blotting (Figure 1D-F). In accordance with the above results, higher expression of PHB2 was observed in the NSCLC cell lines than in the BEAS-2B cell line at both the mRNA and protein levels.

We also evaluated the prognostic value of PHB2 expression in clinical cases using the Kaplan-Meier (KM) Plotter platform (www.kmplot.com). Interestingly, the KM Plotter analysis showed that PHB2 expression was negatively correlated with overall survival (OS) in patients with lung cancer, and a similar result was observed in patients with lung adenocarcinoma (Figure 1G-H).

We next explored the correlation between increased PHB2 expression and clinicopathological parameters of patients with NSCLC to assess the clinical significance (Table S1). Intriguingly, PHB2 expression was significantly correlated with differentiation, clinical stage, and lymph node metastasis, whereas no significant differences were identified in PHB2 expression with respect to patient age and sex. These results indicate that PHB2 might be an independent prognostic factor in patients with NSCLC.

### PHB2 promotes proliferation of NSCLC cells

**Figure 2:**
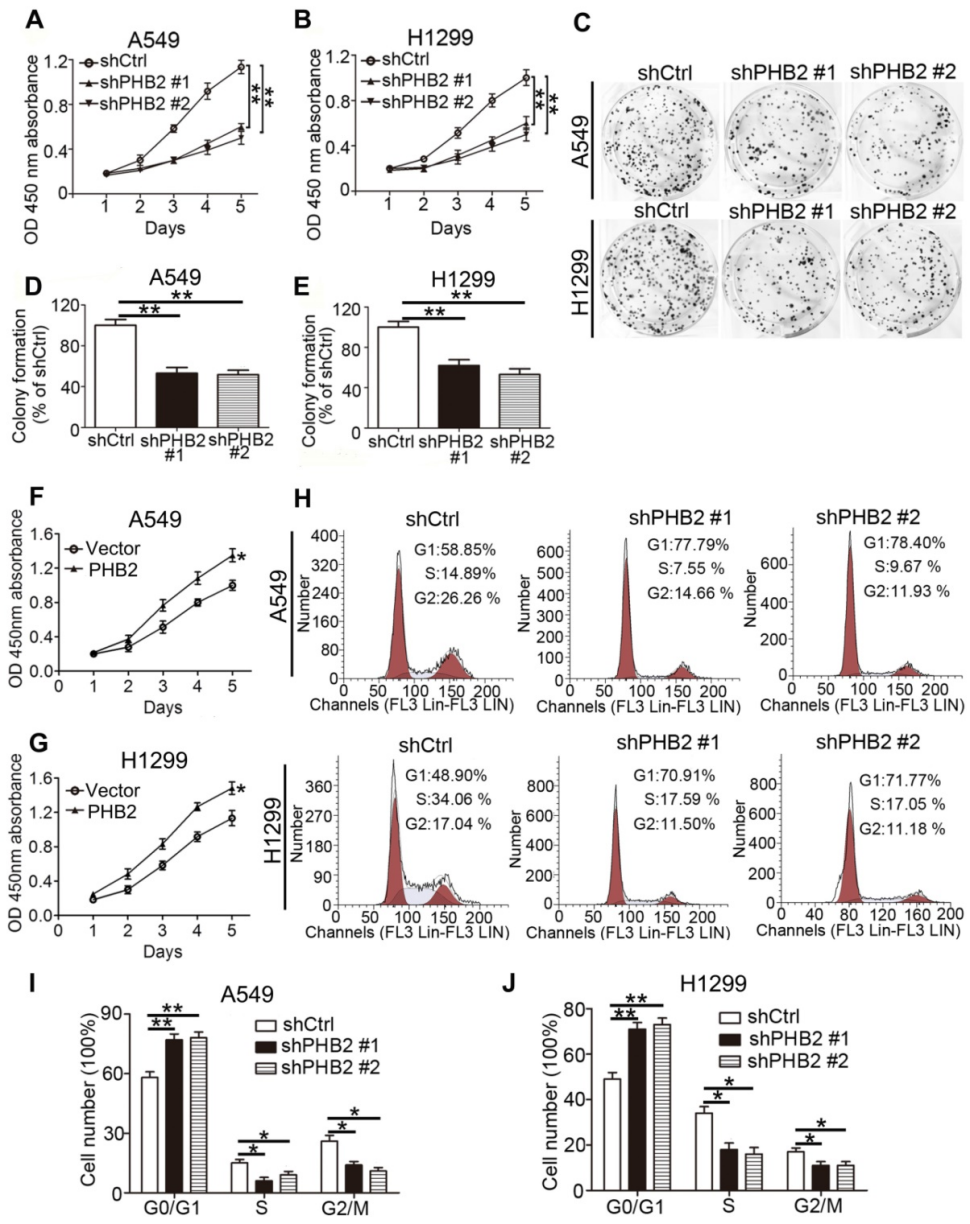
** Effects of PHB2 knockdown and overexpression on proliferation of NSCLC cells.** (A)-(B) Time-dependent cell viability of NSCLC cells with stable PHB2 knockdown measured by CCK-8 assay. (C)-(E) Representative images and quantitation of colony formation of NSCLC cells with stable PHB2 knockdown. (F)-(G) Time-dependent cell viability of NSCLC cells with stable PHB2 overexpression measured by CCK-8 assay. (H)-(J) Representative flow cytometry plots of cell cycle distribution and the percentage of cells in each phase for NSCLC cells with stable PHB2 knockdown. These tests were repeated three times independently. Data are presented as mean ± SEM. *P < 0.05; **P < 0.01. Abbreviations: OD, optical density.

For stable knockdown and overexpression of PHB2, we infected A549 and H1299 cells with lentiviruses. Knockdown and overexpression efficiency at the protein level were confirmed by western blotting (Figure S1). We conducted CCK-8 cell viability, colony formation, and flow cytometry assays to assess the influence of expression changes on cell proliferation. The results show that cell viability was significantly inhibited by PHB2 depletion in A549 and H1299 cells in a time-dependent manner (Figure 2A-B). Colony formation assays showed that PHB2 depletion markedly inhibited the clonogenic growth of A549 and H1299 cells (Figure 2C-E). In contrast, PHB2 overexpression enhanced cell viability (Figure 2F-G) and promoted clonogenic growth of A549 and H1299 cells (Figure S2).

Flow cytometric cell cycle analysis was employed to better explore the inhibitory effect on the cell cycle induced by PHB2 depletion in NSCLC cells (Figure 2H-J). We found that the proportion of cells in the G0/G1 phase was increased in PHB2 knockdown A549 and H1299 cells. Correspondingly, decreased proportions of cells in the S and G2/M phases were observed compared with those in the negative control cells. These data indicate that PHB2 might be a vital regulator of proliferation.

### PHB2 knockdown enhances apoptosis of NSCLC cells

**Figure 3:**
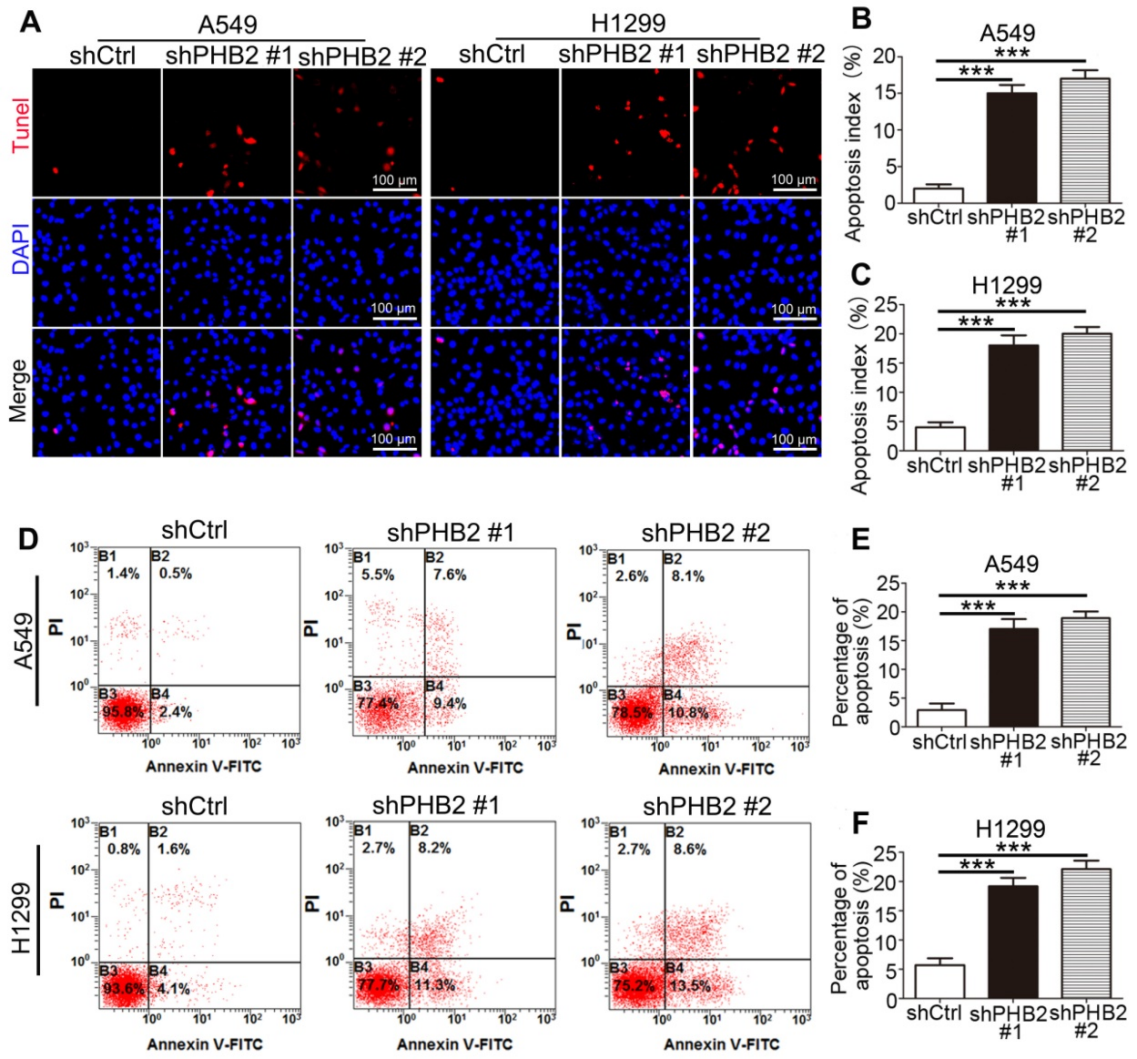
** Effects of PHB2 knockdown on apoptosis of NSCLC cells.** (A)-(C) Representative images of the TUNEL assay and quantitative analysis of the apoptotic index for NSCLC cells with stable PHB2 knockdown. (D)-(F) Representative flow cytometry plots of annexin V and PI double staining and quantification of apoptosis for NSCLC cells with stable PHB2 knockdown. The apoptotic rate was calculated by adding the percentages of the right two quadrants. The proportions of non-apoptotic cells (annexin V-FITC^-^/PI^-^), early apoptotic cells (annexin V-FITC^+^/PI^-^), late apoptotic cells (annexin V-FITC^+^/PI^+^) and dead cells (annexin V-FITC^-^/PI^+^) are indicated. These tests were repeated three times independently. Data are presented as mean ± SEM. ***P < 0.001. Abbreviations: PI, propidium iodide; TUNEL, terminal deoxynucleotidyl transferase dUTP nick end labeling.

It is well known that changes in the balance between proliferation and apoptosis are considered to be closely related to the etiology of cancer. The inhibitory effect of PHB2 depletion on NSCLC cell growth may be partially due to induction of apoptosis. TUNEL assays showed that more TUNEL-positive A549 and H1299 cells were detected in the PHB2-knockdown group than in the negative control group (Figure 3A-C). Similar results were found in the flow cytometric apoptosis analysis, as evidenced by an apparently greater increase in the apoptotic rate in PHB2 knockdown A549 and H1299 cells (Figure 3D-F). These data indicate that PHB2 knockdown promotes apoptosis of NSCLC cells.

### PHB2 promotes migration and invasion of NSCLC cells

**Figure 4:**
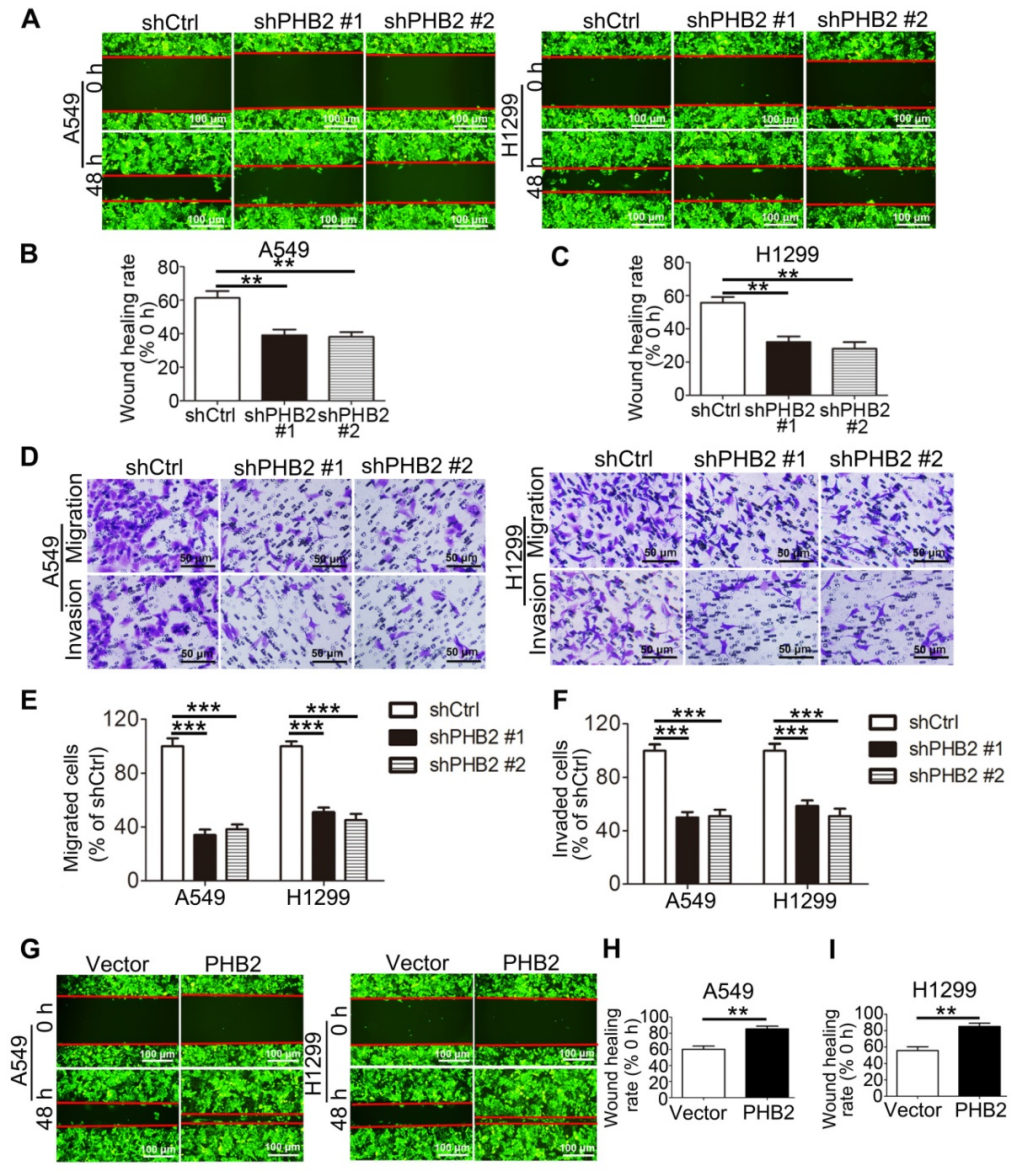
** Effects of PHB2 knockdown and overexpression on migration and invasion of NSCLC cells.** (A)-(C) Representative wound healing photomicrographs and quantitative analysis of wound healing rates at 0 and 48 h of NSCLC cells with stable PHB2 knockdown. (D)-(F) Representative images and quantitative analysis of stained migrated or invaded NSCLC cells with stable PHB2 knockdown. (G)-(I) Representative wound healing photomicrographs and quantitative analysis of wound healing rates at 0 and 48 h of NSCLC cells with stable PHB2 overexpression. These tests were repeated three times independently. Data are presented as mean ± SEM. **P < 0.01; ***P < 0.001.

Because acquisition of an invasive phenotype is an important step in the progression of malignant tumors, we assessed the effect of PHB2 on the mobility of NSCLC cell lines. In scratch assays, PHB2 knockdown delayed wound healing of A549 and H1299 cells (Figure 4A-C). In Transwell assays, consistent with the results of the scratch assays, migratory capability was inhibited in PHB2 knockdown A549 and H1299 cells compared to that in control cells (Figure 4D-E). In contrast, PHB2 overexpression enhanced the migratory capability of NSCLC cells in both the scratch assays and the Transwell assays (Figures 4G-I and S3A-B). Transwell assays were also performed to evaluate the effect of PHB2 on the invasive phenotype. A noticeable reduction in invasive potential was similarly detected with PHB2 depletion (Figure 4D, F), whereas upregulation of PHB2 sharply increased the invasive potential of NSCLC cells (Figure S3A, C). These findings indicate that PHB2 may exert a crucial regulatory influence on the migration and invasion capacities of NSCLC cells.

### PHB2 knockdown inhibits tumorigenesis in vivo

**Figure 5:**
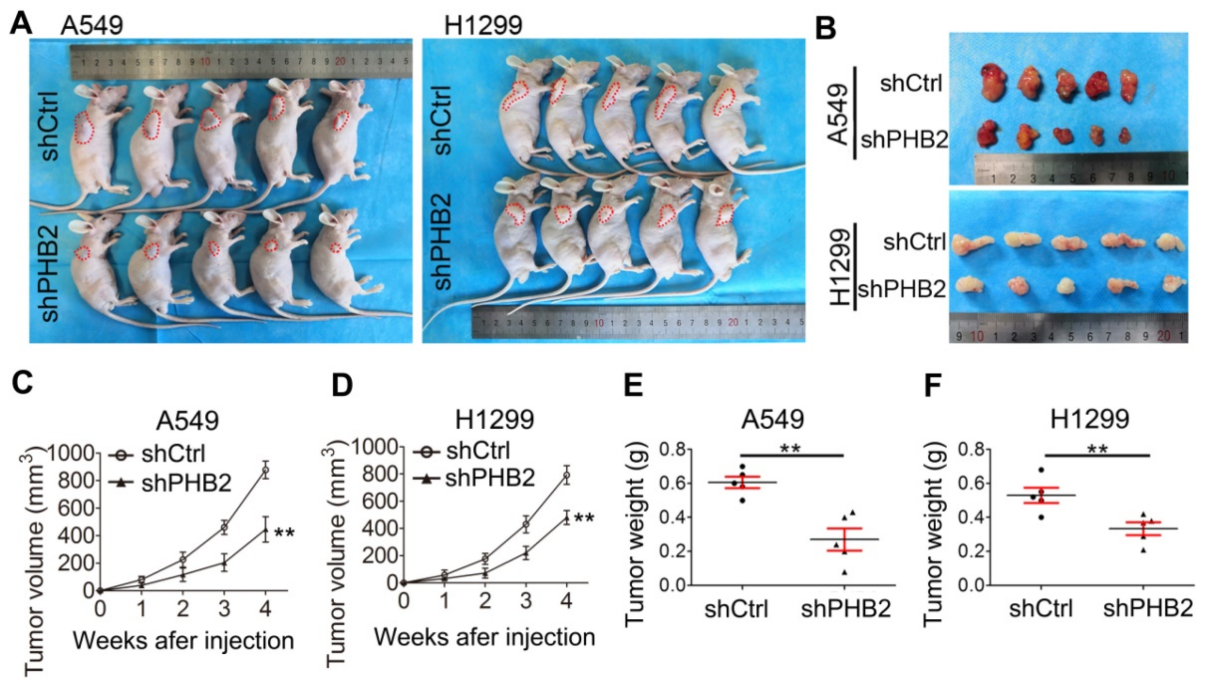
** Effects of PHB2 knockdown on tumorigenesis in vivo.** (A)-(B) Images showing the morphology of NSCLC cell-derived tumor xenografts in each group. (C)-(D) Tumor growth curves showing the growth of PHB2 knockdown and control xenografts in vivo. (E)-(F) Tumor weight of PHB2 knockdown and control xenografts. Data are presented as mean ± SEM. **P < 0.01.

To further validate the effect of PHB2 on tumorigenesis in vivo, a tumor xenograft assay was performed. A549 and H1299 cells with endogenous PHB2 depletion were subcutaneously injected into nude mice (Figure 5A-B), and xenograft tumor growth was monitored periodically. The transplanted tumors were weighed after the nude mice were sacrificed. As expected, we found that PHB2 knockdown led to a marked decrease in xenograft tumor volume and tumor weight (Figure 5C-F). These data indicate that PHB2 knockdown inhibits tumorigenesis in vivo.

### PHB2 interacts with RACK1 and regulates the stability of RACK1 in A549 cells

**Figure 6:**
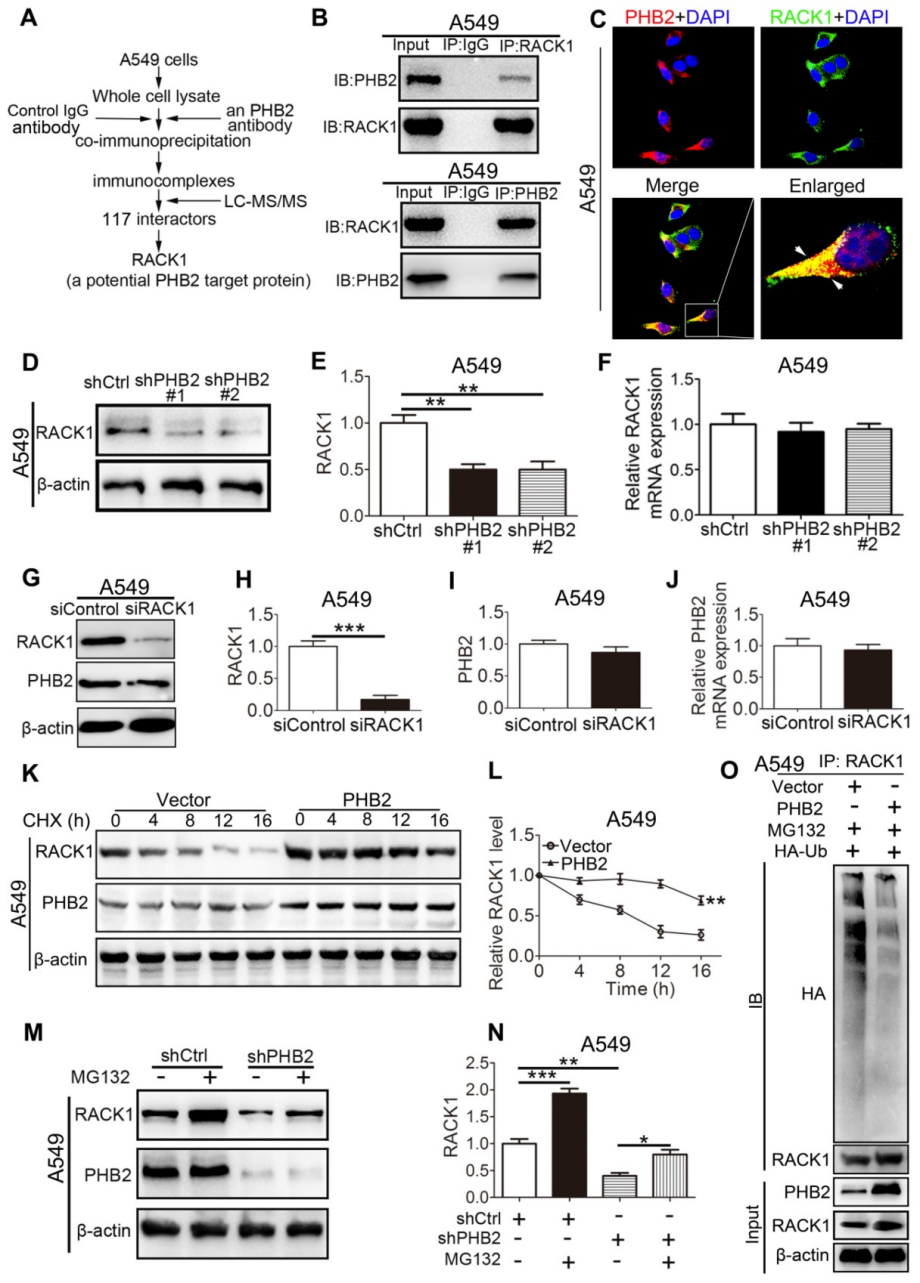
** PHB2 interacts with RACK1 and regulates the stability of RACK1 in A549 cells.** (A) Flowchart to identify proteins interacting with PHB2. Whole A549 cell lysates were prepared for immunoprecipitation using an anti-PHB2 antibody or a control IgG antibody, and the immunocomplexes were analyzed using LC-MS/MS. A total of 117 potential interacting proteins with high fidelity were identified. RACK1 was identified as a potential PHB2 target protein. (B) Endogenous Co-IP assays between PHB2 and RACK1 in A549 cells. (C) Representative immunofluorescence images of PHB2 and RACK1 colocalization in A549 cells. (D)-(E) Representative immunoblots for RACK1 and β-actin and their densitometric quantification in A549 cells with stable PHB2 knockdown. (F) mRNA level of RACK1 in A549 cells with stable PHB2 knockdown. (G)-(I) Representative immunoblots for RACK1, PHB2, and β-actin and their densitometric quantification in A549 cells transfected for 48 h with a specific siRNA targeting RACK1. Western blot confirming RACK1 knockdown efficiency at the protein level. (J) mRNA level of PHB2 in A549 cells with RACK1 inhibition. (K) Representative immunoblots for RACK1, PHB2, and β-actin in A549 cells with stable PHB2 overexpression treated with CHX (a protein synthesis inhibitor, 10 µg/mL) at the indicated time points. (L) Evaluation of the half-life of RACK1. (M)-(N) Representative immunoblots for RACK1, PHB2, and β-actin and their densitometric quantification in A549 cells with stable PHB2 knockdown treated with MG132 (a proteasome inhibitor, 10 μΜ) for 8 h before collection. (O) Immunoprecipitation with RACK1 antibody and western blotting with anti-HA antibody to detect ubiquitinated RACK1 in whole-cell lysates of A549 cells with or without PHB2 overexpression transfected with HA-Ub and then treated with MG132 (10 μΜ) for 8 h before collection. These tests were repeated three times independently. Data are presented as mean ± SEM. *P < 0.05; **P < 0.01; ***P < 0.001. Abbreviations: CHX, cycloheximide; Co-IP, coimmunoprecipitation; DAPI, 4',6-diamidino-2-phenylindole; LC-MS/MS, liquid chromatography and tandem mass spectrometry; siRNA, small interfering RNA.

To uncover the molecular mechanism through which PHB2 regulates the NSCLC cell phenotype, we aimed to identify potential proteins that interact with PHB2. Whole A549 cell lysates were prepared for immunoprecipitation using an anti-PHB2 antibody or a control IgG antibody, and immunocomplexes were analyzed using LC-MS/MS (Figures 6A and S4). A total of 117 potential interacting proteins with high fidelity were identified (Tables S2-3). Among them, RACK1 plays crucial roles in tumorigenesis, so we selected this protein for further investigation. To further confirm the interaction between PHB2 and its putative binding partner RACK1, endogenous Co-IP assays were performed in A549 cells and interaction was detected with an anti-RACK1 antibody (Figure 6B). Consistently, a reciprocal Co-IP assay using an anti-PHB2 antibody also confirmed the association between PHB2 and RACK1 (Figure 6B). Next, we employed immunofluorescence analysis to detect PHB2 and RACK1 in A549 cells. PHB2 and RACK1 were found to colocalize in A549 cells (Figure 6C). These data indicate that PHB2 interacts with RACK1 in A549 cells and that the interaction of PHB2 with RACK1 may regulate tumorigenesis in NSCLC.

To explore the possible regulatory effect of PHB2 on RACK1, we detected the expression of RACK1 in A549 cells with stable knockdown or overexpression of PHB2 by western blotting and qRT-PCR (Figures 6D-F and S5). An apparently increased RACK1 protein level was observed in PHB2-overexpressing cells, whereas significantly decreased RACK1 protein expression was detected in PHB2-knockdown cells compared with control cells (Figures 6D-E and S5A-B). Interestingly, there was no significant change in RACK1 mRNA levels in A549 cells with either stable knockdown or overexpression of PHB2 (Figures 6F and S5C). In contrast, knockdown of RACK1 specifically reduced RACK1 expression with no effect on PHB2 mRNA and protein expression (Figure 6G-J). Therefore, PHB2 may regulate RACK1 expression through posttranslational modification.

To further examine whether PHB2 regulates the stability of RACK1, we treated A549 cells with cycloheximide (CHX, an inhibitor of protein synthesis) (Figure 6K-L). Remarkably, PHB2 overexpression prolonged the half-life of endogenous RACK1 protein. In addition, to test whether PHB2 regulates the degradation of RACK1 in a proteasome-dependent manner, A549 cells were treated with the proteasome inhibitor MG132 (Figure 6M-N). As expected, downregulation of RACK1 protein was evident with PHB2 depletion and was blocked by MG132, which interrupts the ubiquitin-proteasome pathway. Previous studies reported that RACK1 turnover is influenced by the ubiquitin-proteasome system 29, 30. As PHB2 binds to RACK1 and positively regulates RACK1 protein stability, we hypothesized that PHB2 increases RACK1 expression by decreasing ubiquitination of RACK1. To address this hypothesis, we analyzed the ubiquitination of RACK1. As expected, RACK1 could be ubiquitinated in lung cancer cells, and PHB2 overexpression reduced RACK1 ubiquitination (Figure 6O).

### PHB2 promotes tumorigenesis by regulating RACK1 and RACK1-associated proteins as well as downstream signaling in vitro

**Figure 7:**
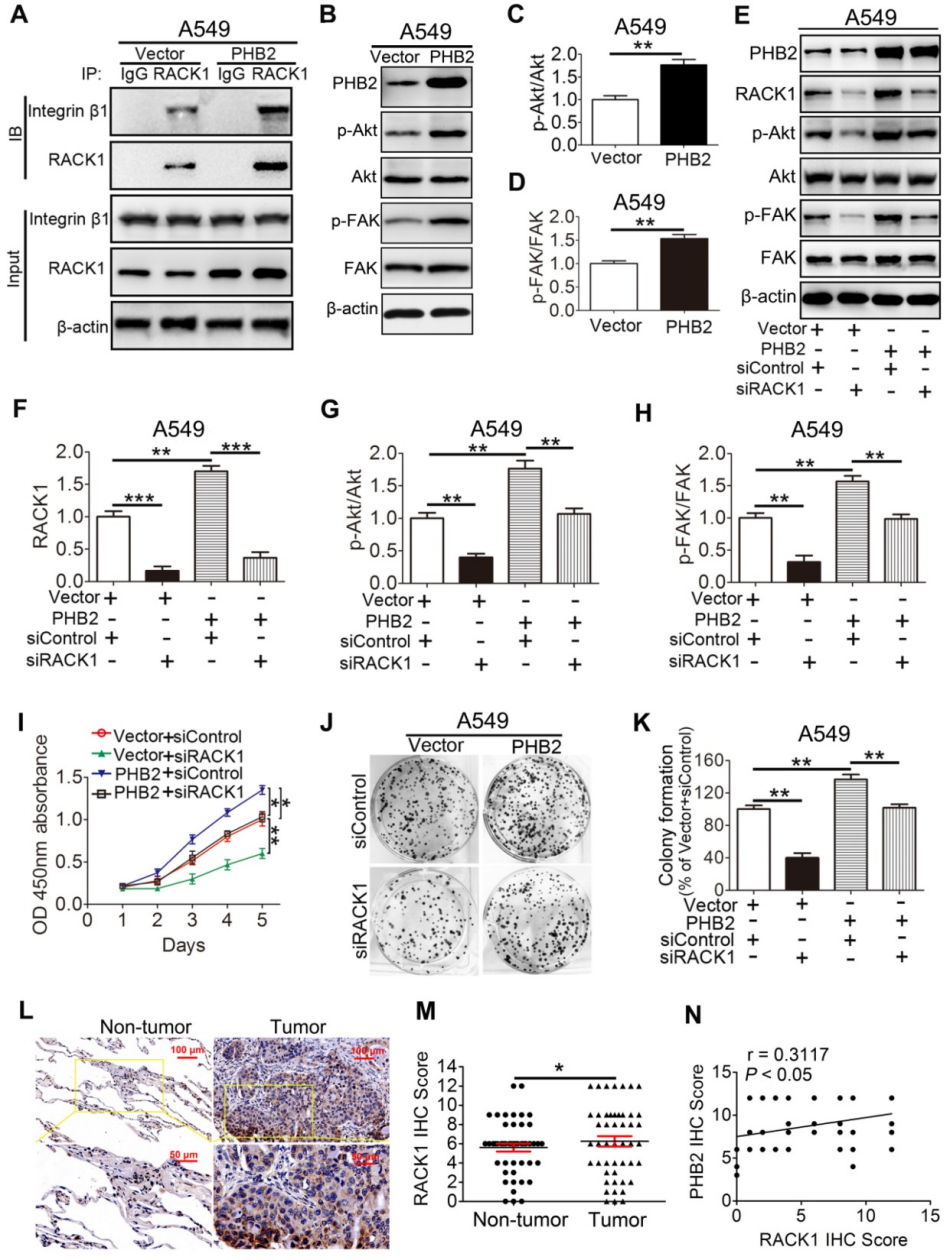
** PHB2 promotes tumorigenesis by regulating RACK1 and RACK1-associated proteins and downstream signaling in vitro.** (A) Endogenous Co-IP assays between RACK1 and integrin β1 using an anti-RACK1 antibody in A549 cells with stable PHB2 overexpression. (B)-(D) Representative immunoblots for PHB2, p-Akt, Akt, p-FAK, FAK, and β-actin and their densitometric quantification in A549 cells with stable PHB2 overexpression. (E)-(H) Representative immunoblots for PHB2, RACK1, p-Akt, Akt, p-FAK, FAK, and β-actin and their densitometric quantification in A549 cells with stable PHB2 overexpression transfected with a specific siRNA targeting RACK1. (I) Time-dependent cell viability of A549 cells from the indicated groups measured by CCK-8 assay. (J)-(K) Representative images and quantitation of colony formation of A549 cells from the indicated groups. These tests were repeated three times independently. (L) Representative images of IHC staining for RACK1 in 48 paired NSCLC tumor and adjacent normal tissues. (M) Quantitative analysis of RACK1 protein expression in tumor and tumor-adjacent tissues. (N) Correlation between the protein levels of RACK1 and PHB2 in 48 NSCLC tissues calculated using Pearson's correlation test (r = 0.3117, *P <* 0.05, n = 48). Data are presented as mean ± SEM. *P < 0.05; **P < 0.01; ***P < 0.001. Abbreviations: IHC, immunohistochemistry; siRNA, small interfering RNA.

Our findings demonstrate that PHB2 interacts with RACK1 and affects its protein expression in NSCLC cells. However, whether PHB2 functions through RACK1 is largely unknown. Increasing evidence indicates that RACK1 adopts a seven-bladed β-propeller structure that facilitates protein binding 15. Notably, RACK1 can bind to integrin β1 and manipulate the activity of integrin and integrin receptor complexes and downstream intracellular signaling pathways, including the Akt and FAK signaling pathways 15, 31, 32. We found that RACK1 interacts with integrin β1 in A549 cells (Figure 7A), which is consistent with previous literature 15, 31, 32. Moreover, an increased amount of integrin β1 was pulled down by RACK1 in A549 cells stably overexpressing PHB2 compared with negative control cells (Figure 7A). To determine whether the increased amount of integrin β1 pulled down by RACK1 is facilitated by PHB2 overexpression or is an indirect effect of increased stability of the RACK1 protein, the proteasome inhibitor MG132 was employed. Interestingly, MG132 treatment also increased the amount of integrin β1 pulled down by RACK1, indicating that the enhanced level of integrin β1 pulled down by RACK1 may be induced by the increased stability of the RACK1 protein (Figure S6). Remarkably, PHB2 overexpression also resulted in activation of the Akt and FAK pathways (Figure 7B-D). These findings suggest that PHB2 regulates RACK1 and RACK1-associated proteins as well as downstream signaling in A549 cells.

To further explore whether PHB2 promotes tumor progression by regulating RACK1, we depleted endogenous RACK1 in PHB2-overexpressing A549 cells (Figure 7E-F). RACK1 inhibition abolished PHB2-mediated activation of the Akt and FAK pathways (Figure 7E-H). As expected, depletion of endogenous RACK1 expression also reversed PHB2-induced cell growth, migration, and invasion (Figures 7I-K and S7A-C). In addition, RACK1 expression in 48 pairs of human clinical NSCLC tissues and their corresponding noncancerous lung tissues was examined by IHC (Figure 7L). RACK1 expression in NSCLC was significantly higher than that in adjacent noncancerous lung tissues (Figure 7M). Notably, statistical analysis showed a significant positive correlation between PHB2 and RACK1 (Figure 7N). These data indicate that PHB2 promotes tumorigenesis in A549 cells by regulating RACK1 and RACK1-associated proteins as well as downstream signaling.

## Discussion

**Figure 8:**
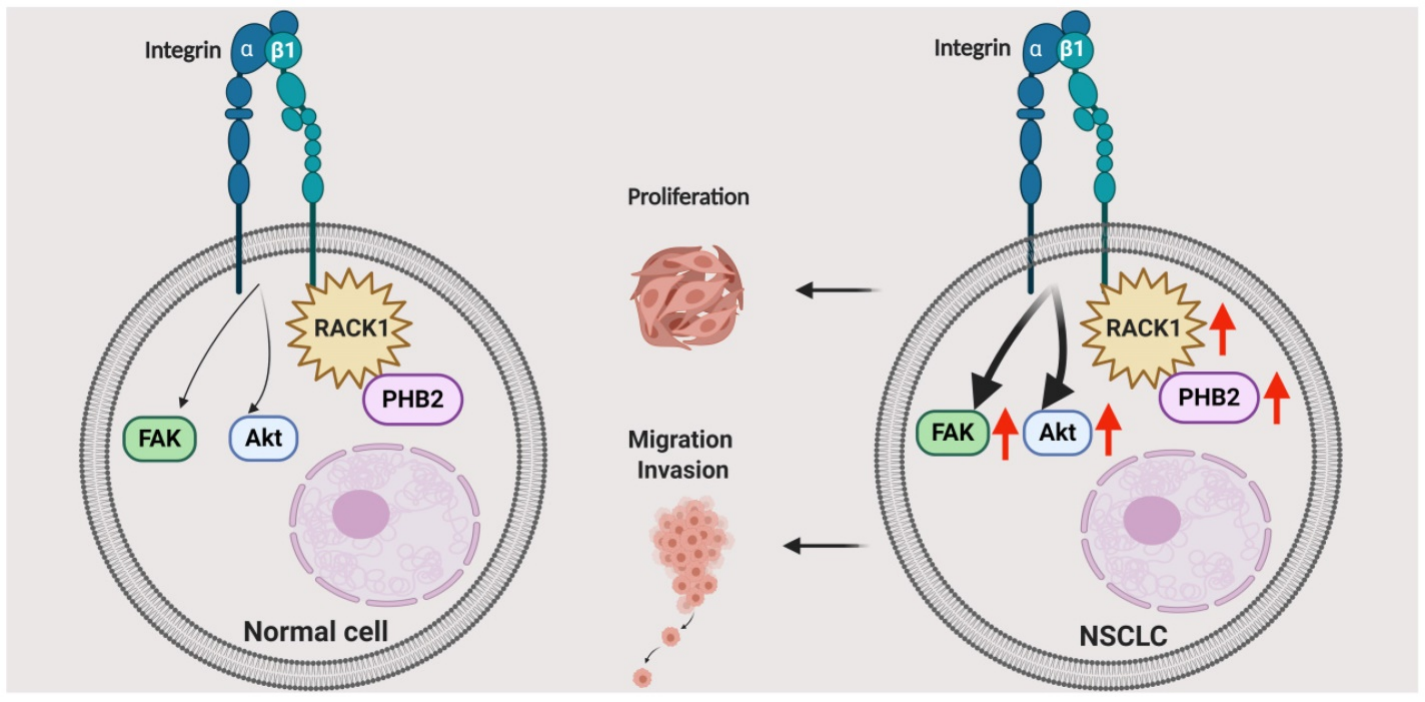
** Schematic illustrating the role of PHB2 in regulating NSCLC tumorigenesis.** PHB2 is a pivotal tumorigenic factor involved in the cellular processes of proliferation, apoptosis, migration, and invasion of NSCLC cells. PHB2 interacts with RACK1 and increases RACK1 protein expression, which further upregulates the amount of integrin β1 pulled down by RACK1 and induces activation of the Akt and FAK pathways in NSCLC cells. The crosstalk between PHB2 and RACK1 may play an important role in regulating tumorigenesis in NSCLC.

PHB2 is a highly evolutionarily conserved protein that is involved in various physiological processes regulating cell differentiation, transcriptional modification, nuclear signaling, mitochondrial dynamics coordination, and mitophagy in various cellular compartments 33-36. PHB2 can regulate multiple pathologies such as cancer, neuromuscular degeneration, and other metabolic diseases 34, 36. However, most of the current knowledge concerning PHB proteins in cancers has been gleaned from studies with prohibitin 1 (PHB1), while studies on the effects of PHB2 have been limited to date, especially in NSCLC 34, 37-40. Our major finding in the present study is that PHB2 facilitates tumorigenesis in NSCLC by interacting and stabilizing RACK1, which further induces changes in downstream tumor effectors and pathways (Figure 8). We found that PHB2 is overexpressed in NSCLC tumor tissues compared to normal controls and that its expression is correlated with clinicopathological features. Furthermore, PHB2 promotes proliferation, migration, and invasion, whereas PHB2 knockdown enhances the rate of apoptosis in NSCLC cells. Moreover, PHB2 interacts and stabilizes RACK1 through posttranslational modification, which further induces activation of the Akt and FAK pathways. The stimulative effect of PHB2 on tumorigenesis was also verified in vivo. Therefore, our results reveal the role of PHB2 in tumorigenesis and the regulation of RACK1 stability in NSCLC.

As a contributing factor in other cancer types, PHB2 has been reported to regulate multiple processes via different pathways involved in tumorigenesis 37. Cheng *et al.* 11 found significant upregulation of PHB2 in hepatocellular carcinoma tissues. They argued that PHB2 knockdown suppresses cell growth and colony formation, leads to G1 phase arrest, sensitizes tumor cells to apoptosis, and inhibits the ability of tumor cells to adapt to hypoxic microenvironments. Kasashima *et al.* 41 found that PHB2 had a similar effect on apoptosis in HeLa cells, and they reported that PHB2 knockdown resulted in caspase-dependent apoptosis through Hax-1 downregulation and mitochondrial fragmentation. Zhou *et al.* 42 suggested the protumorigenic role of PHB2 in human rhabdomyosarcoma via manipulation of its localization and regulation of proliferation and apoptosis. Shen *et al.* 10 showed that PHB2 promoted prostate cancer cell migration by regulating Akt2 expression and stability. A recent study 43 reported that PHB2 knockdown inhibited proliferation, migration, and invasion in NSCLC cells by regulating mitophagy, but the exact role and underlying mechanism of PHB2 in NSCLC still need to be explored. In our study, we found that PHB2 expression in NSCLC was significantly higher than that in adjacent noncancerous lung tissues. Similar changes in PHB2 protein expression were also observed when comparing NSCLC cells and the normal human bronchial epithelial cell line BEAS-2B. What is noteworthy is that overexpression of PHB2 promoted tumorigenesis in NSCLC cells, while deficiency of PHB2 suppressed the tumorigenesis of NSCLC in vitro and in vivo, which is similar to the effect of PHB2 on tumorigenesis in other tumor types 10, 11, 41, 42. Notably, the effects of PHB2 on tumor proliferation are different in estrogen receptor (ER)-positive breast cancer. PHB2 exerts a repressive effect on estrogen-dependent transcriptional activity and inhibits the growth of breast cancer 44, 45. These completely diverse effects on cell proliferation may be due to the multiple cellular functions of PHB2. It is well known that PHB2 presents clear and distinctive functions depending on its intracellular localization in different cell types 34, 36. In breast cancer, the translocation of PHB2 to the nucleus leads to transcriptional suppression of ERα 44, 45. However, in the present study, PHB2 was found to be diffused mainly throughout the membrane and cytoplasm in NSCLC cells according to both IHC staining and immunofluorescence analysis. Combined with previous studies on PHB2 in other cancer types, we speculate that PHB2 may exert its influence on tumorigenesis in NSCLC based on its intracellular localization, and new mechanisms may be implicated in the future.

In the present study, we used LC-MS/MS to identify potential proteins interacting with PHB2 and identified a potential target, RACK1. Co-IP assays and immunofluorescence staining also confirmed the interaction between PHB2 and RACK1. RACK1, regarded as a multifaceted protein, is a key mediator of various pathways and contributes to numerous aspects of cellular function 15. Aberrant expression of RACK1 has been observed in many cancer types, where it acts as a tumor promoter or suppressor in a tissue-type and context-dependent manner 17-23, 46. Notably, RACK1 has been validated as an oncogene in NSCLC 21, 22. In our study, we observed increased RACK1 protein levels in PHB2-overexpressing cells but decreased RACK1 protein expression in PHB2-knockdown cells compared to control cells, suggesting that PHB2 promotes tumorigenesis via RACK1 in NSCLC. In addition, although PHB2 has been reported to interact with some transcription factors and modulate the transcription of some genes 15, 47-49, no significant change in RACK1 mRNA levels was observed in A549 cells with either stable knockdown or overexpression of PHB2, indicating that PHB2 regulates RACK1 expression through posttranslational modification. Consistently, we found that PHB2 overexpression prolonged the half-life of the RACK1 protein. In addition, PHB2 decreased degradation of RACK1 in a proteasome-dependent manner, as evidenced by the restrained effect of the proteasome inhibitor MG132 on RACK1 degradation and the decreased RACK1 ubiquitination in PHB2-overexpressing cells. Although Day *et al.* 29 identified RAB40C as a ubiquitin E3 ligase responsible for the ubiquitination of RACK1, other E3 ligases may also be involved in RACK1 ubiquitination, and this issue deserves deeper exploration. All these findings indicate that PHB2 may be involved in the modulation of RACK1 stability and expression via posttranslational modification.

Numerous studies have verified that RACK1 can scaffold other target proteins, kinases, and phosphatases and alter the activity of several of these proteins in cancer 15, 16, 50, 51. In particular, altered RACK1 expression (either up or down) has dramatic and distinct effects on the properties and activities of its downstream binding partners, the regulation of some key pathways, and the development and progression of neoplastic disease 15, 50. It is worth noting that RACK1 can interact with integrin β1, alter the activity of integrin, and further activate downstream intracellular signaling pathways, including Akt and FAK signaling 15, 50, 52-54. In the present study, consistent with previous studies, PHB2 overexpression enhanced the amount of integrin β1 pulled down by RACK1 and led to activation of the Akt and FAK pathways in A549 cells. Furthermore, as expected, RACK1 depletion reversed the promotive effect of PHB2 on the Akt and FAK pathways and abolished the effect of PHB2 on tumorigenesis in A549 cells. These findings indicate that PHB2 facilitates tumorigenesis by regulating RACK1 and RACK-associated downstream proteins and signaling. Importantly, previous studies have demonstrated that PHB2 can be phosphorylated by many kinases, including Akt 55. Moreover, a study reported that phosphorylation of PHB2 on serine 91 (S91) results in a rapid loss of viability and apoptotic cell death and serves to coordinate nuclear-mitochondrial events in promyelocytic leukemia cells during differentiation 56. Whether PHB2 can be phosphorylated by Akt in NSCLC cells remains unknown. Given the complexity of signal regulation, one possibility is that PHB2-RACK1 regulation is reciprocal. Further study is warranted to test this possibility. Since RACK1 mediates physical interactions with a myriad of signaling proteins 16, we cannot rule out the possibility that RACK1 has many other biological effects and is associated with multiple molecules and signaling pathways. The precise role of RACK1 in NSCLC deserves further investigation.

However, there are limitations to this study. First, some of the conclusions in this study were not verified in vivo. Second, the interaction between PHB2 and RACK1 may be based on a unique structure, but the exact binding mechanism was not elucidated in our study and needs to be explored further. Third, this PHB2-RACK1 regulation might be generally applicable to other cancer types. However, this speculation still needs to be fully investigated. In addition, PHB2 and its homologous protein PHB1 are functionally interdependent 14, but whether the effect of PHB1 in NSCLC is also related to RACK1 remains unknown. Despite these limitations, we believe that our findings provide important new insights for understanding the role of PHB2 in tumorigenesis in NSCLC and suggest that PHB2 could serve as a potential molecular target for the future development of anti-NSCLC therapy.

In summary, we identified the multifunctional protein PHB2 as a pivotal tumorigenic factor involved in the cellular processes of proliferation, cell cycle arrest, apoptosis, migration, and invasion in NSCLC cells (Figure 8). PHB2 was also identified as a novel regulator of RACK1 that positively modulates its stability and activity, further increasing the amount of integrin β1 pulled down by RACK1 and inducing activation of the Akt and FAK pathways. This cellular study was also partly validated in a tumor xenograft model. Although exactly how PHB2 interacts with RACK1 and how PHB2 regulates its stability remain to be investigated, our study provides new evidence to suggest that PHB2 acts as an oncogenic player in NSCLC. We believe that novel therapeutic strategies for NSCLC can be designed by targeting the signaling mediated by the crosstalk between PHB2 and RACK1.

## Supplementary Material

Supplementary information, figures and tables.

## Abbreviations

CHX: cycloheximide
CCK-8: Cell Counting Kit-8
Co-IP: coimmunoprecipitation
DAB: 3,3'-diaminobenzidine
DAPI: 4',6-diamidino-2-phenylindole
HRP: horseradish peroxidase
IHC: immunohistochemistry
KM: Kaplan-Meier
LC-MS/MS: liquid chromatography and tandem mass spectrometry
NSCLC: non-small-cell lung cancer
OD: optical density
OS: overall survival
PI: propidium iodide
PE: phycoerythrin
PHB2: prohibitin 2
RACK1: receptor for activated C kinase 1
shRNA: short hairpin RNA
siRNA: small interfering RNA
SCLC: small cell lung cancer
TUNEL: terminal deoxynucleotidyl transferase dUTP nick end labeling
