# Supplementary material for: PHB2 promotes tumorigenesis via RACK1 in non-small cell lung cancer
Source: Theranostics. 2021 Jan 1;11(7):3150–66. doi: 10.7150/thno.52848 (PMC7847695; doi:10.7150/thno.52848)
Supplement: Supplementary file 1 — Supplementary information, figures and tables. [file thnov11p3150s1.pdf]

PHB2 promotes tumorigenesis *via* RACK1 in non-small cell lung cancer

Supplemental Material

In-gel digestion and Mass spectrometry analysis

PHB2 proteins were immunoprecipitated with Protein A/G agarose from A549 cells. Anti-IgG was used as a negative control. Purified PHB2 proteins from A549 cells were separated by 7.5% SDS-PAGE and visualized by staining with Coomassie Brilliant Blue (CBB) G-250. The PHB2 protein band or control IgG was directly cut out of gels, destained with 50% acetonitrile in 50 mM ammonium bicarbonate, then dried in a speed vacuum concentrator. Gel pieces were reduced with 5 mg/mL dithiothreitol (DTT) in 50 mM ammonium bicarbonate at 60 ℃ for 1 h, alkylated by 10 mg/mL iodoacetamide in 50 mM ammonium bicarbonate at room temperature for 1 h, then dried in a speed vacuum concentrator. Dried gel pieces were rehydrated with 50 mM ammonium bicarbonate containing 100 μg/mL trypsin and incubated at 37 ℃ for 24 h. Supernatant peptide mixtures were extracted with 50% ammonium bicarbonate in 5% formic acid for 30 min and dried in a speed vacuum concentrator.

A Q-Exactive HF MS (Thermo Fisher Scientific) interfaced with an EasynLC 1200 nanoflow LC system (Thermo Fisher Scientific) was employed to measure the samples from in-gel digestion. Then, raw files were searched against the human refseq protein database with MS analysis. The mass tolerance of the precursor ions and QE HF was set to 20 p.p.m and 50 mmu, respectively. Protease digestion cannot exceed two missed cleavages. The minimal required peptide length was seven amino acids. The data were also searched against a decoy database so that protein identifications were accepted at an FDR of 1%. Protein identification data are available in Tables S2 and S3. Potential PHB2 target proteins should separate false positive proteins that were identified by the control IgG antibody from true positive proteins that were identified with the PHB2 antibody. Three replicates of this analysis were undertaken.

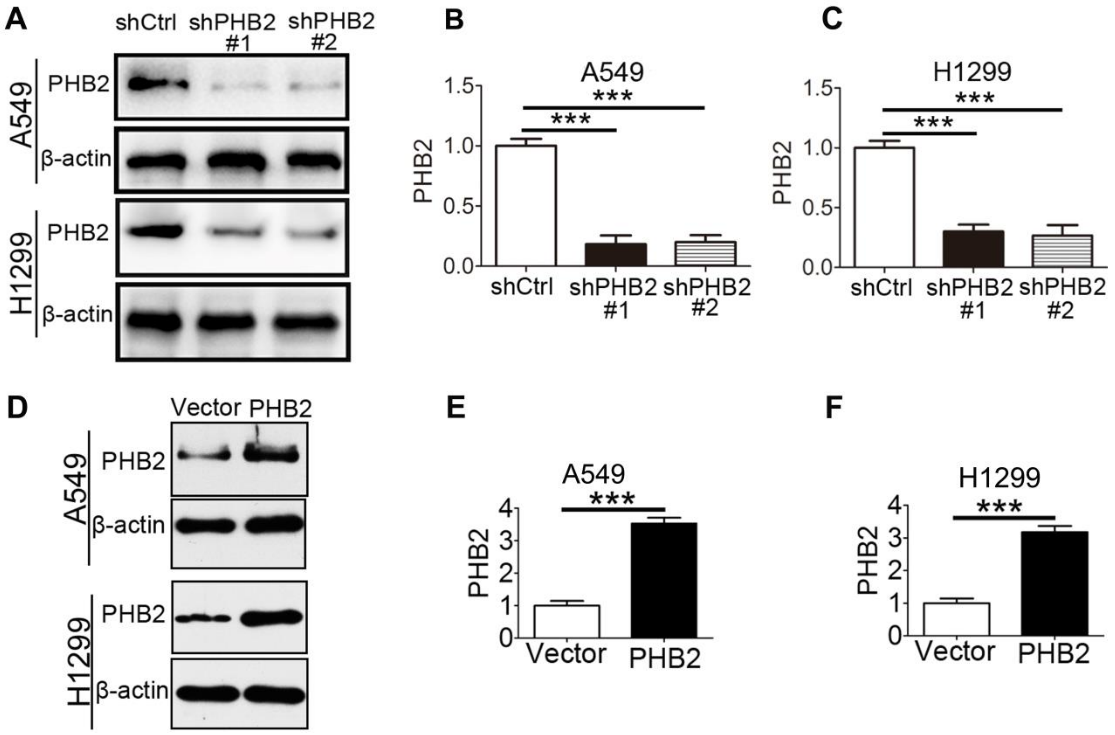

Figure S1. PHB2 knockdown and overexpression efficiency at the protein level were confirmed by western blotting. (A)–(F) Representative immunoblots and densitometric quantification for PHB2 and β-actin in NSCLC cells with stable knockdown or overexpression of PHB2. These tests were repeated three times independently. Data are presented as the mean ± SEM. $***P < 0.001$.

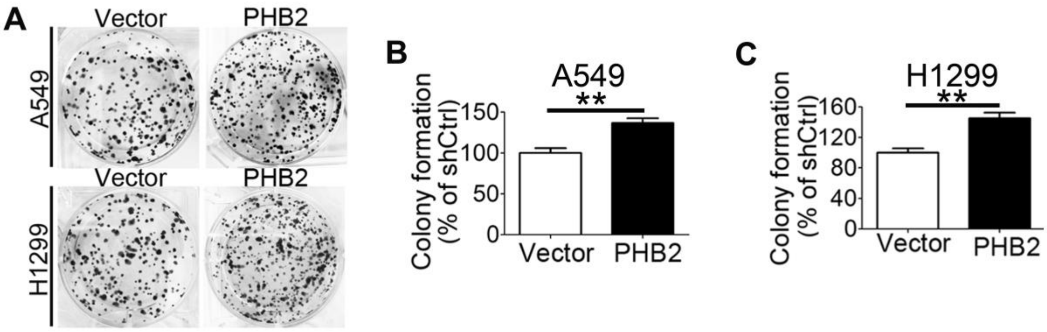

Figure S2. Effects of PHB2 overexpression on colony formation in NSCLC cells. (A)–(C) Representative images and quantitation of colony formation of NSCLC cells with stable PHB2 overexpression. These tests were repeated three times independently. Data are presented as the mean ± SEM. $**P < 0.01$.

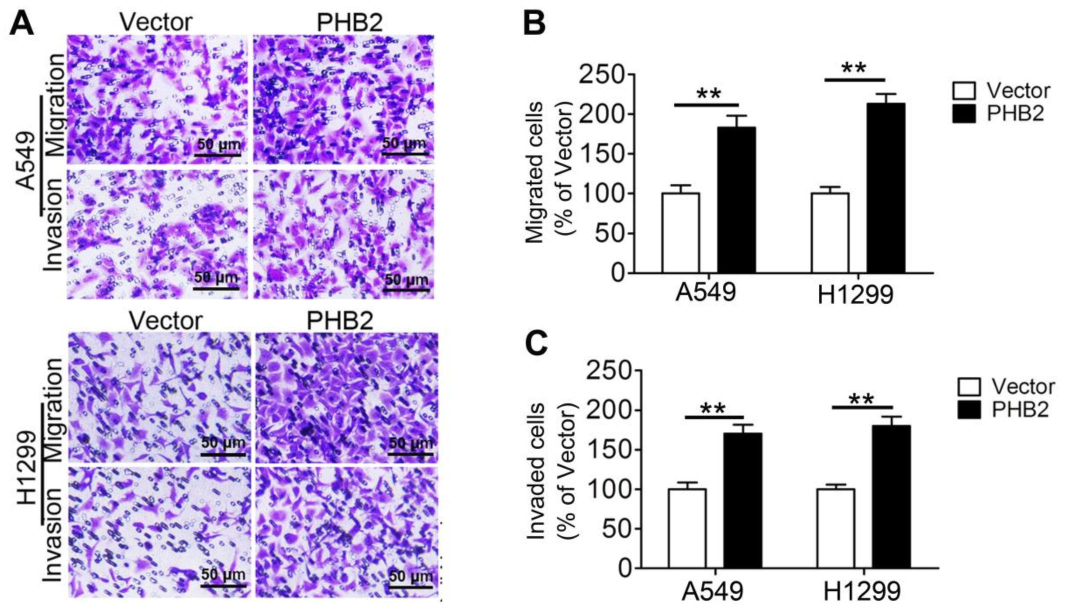

Figure S3. Effect of PHB2 overexpression on migration and invasion in NSCLC cells. (A)–(C) Representative images and quantitative analysis of stained migrated or invaded NSCLC cells with stable PHB2 overexpression. These tests were repeated three times independently. Data are presented as the mean ± SEM. $^{**}P < 0.01$.

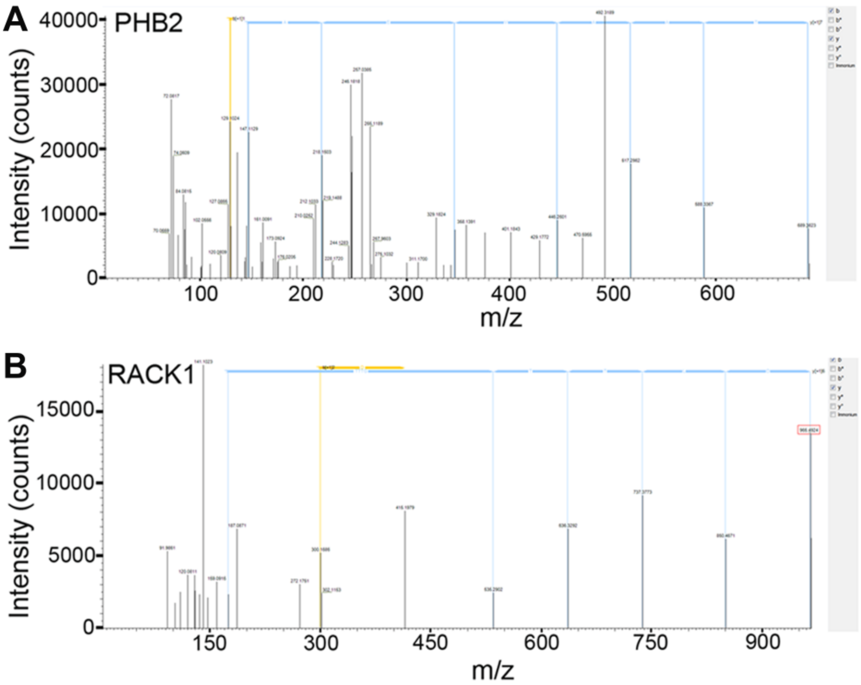

Figure S4. The spectra of PHB2 and RACK1 obtained by LC-MS/MS. (A)–(B) Whole A549 cell lysates were prepared for immunoprecipitation using an anti-PHB2 antibody or a control IgG antibody, and immunocomplexes were analyzed using LC-MS/MS. Three replicates of this analysis were undertaken.

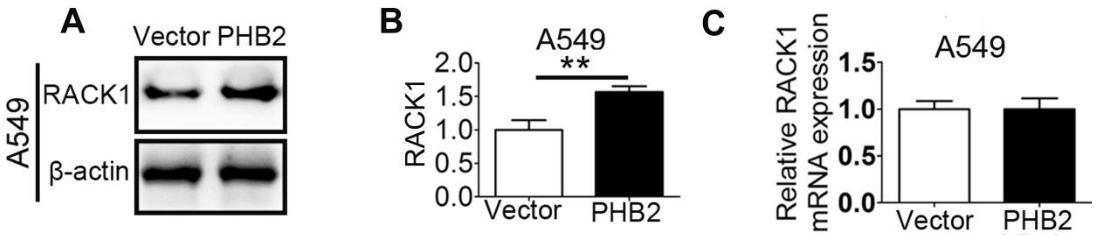

Figure S5. Effects of PHB2 overexpression on RACK1 expression at the protein and mRNA levels. (A)–(B) Representative immunoblots for RACK1 and β-actin and their densitometric quantification in A549 cells with stable PHB2 overexpression. (C) mRNA level of RACK1 in A549 cells with stable PHB2 overexpression. These tests were repeated three times independently. Data are presented as the mean ± SEM. **P < 0.01.

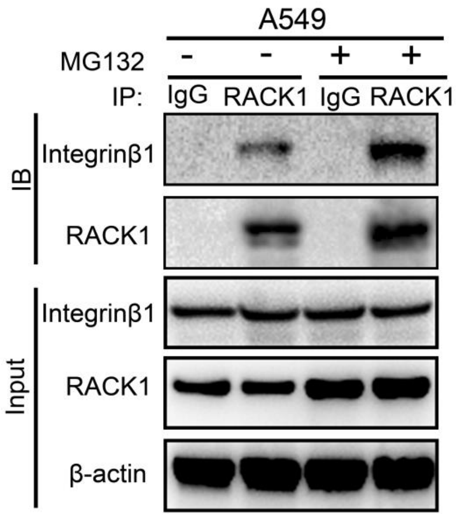

Figure S6. MG132 enhanced the amount of integrin β1 pulled down by RACK1. Endogenous Co-IP assays between RACK1 and integrin β1 using an anti-RACK1 antibody in A549 cells in the absence or presence of MG132 (a proteasome inhibitor). A549 cells were treated with MG132 (10 μM) for 8 h before collection. These tests were repeated three times independently.

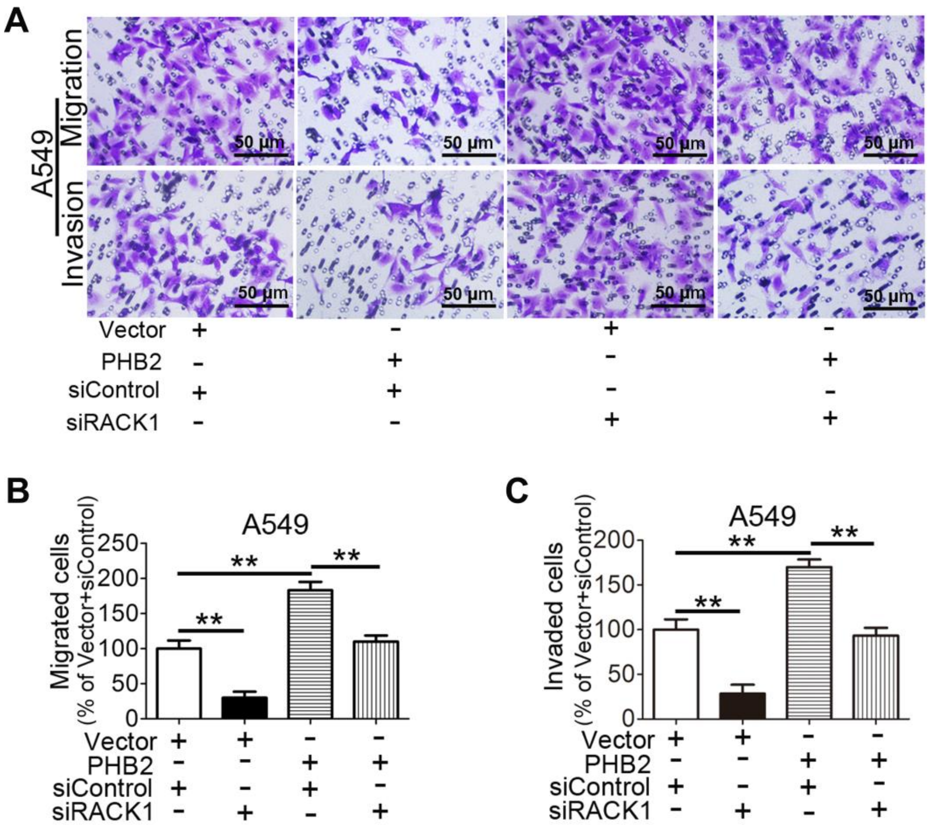

Figure S7. Depletion of endogenous RACK1 expression reversed the effect of PHB2 on the mobility of A549 cells. (A)–(C) Representative images and quantitative analysis of stained migrated cells or invaded A549 cells from the indicated groups. These tests were repeated three times independently. Data are presented as the mean ± SEM. $^{**}P < 0.01$.

Table S1. Association of PHB2 expression with clinicopathological parameters of patients with non-small cell lung cancer.

Clinicopathological variables	n	PHB2 expression		P value
		low	high	
Age				0.6337
<60	27	16	11	
≥60	21	11	10	
Gender				0.5360
Male	25	13	12	
Female	23	14	9	
Differentiation				0.0041**
Well	4	4	1	
moderately	29	20	9	
Poorly	15	3	12	
Lymph node metastasis				0.0309*
Absent	33	22	11	
Present	15	5	10	
Clinical stage				0.0445*
I / II	41	26	15	
III / IV	7	1	6	

*$P < 0.05$; **$P < 0.01$

Table S2. PHB2 interacting partners were identified with LC-MS/MS.

Accession	Description	MW [kDa]	Peptides (n)	Coverage [%]	Sum PEP Score
P04264	Keratin, type II cytoskeletal 1	66	34	51	122.613
P35527	Keratin, type I cytoskeletal 9	62	20	52	76.445
P35232	Prohibitin	29.8	17	76	71.364
P07355	Annexin A2	38.6	17	57	67.083
Q99623	Prohibitin-2	33.3	16	65	58.172
P04406	Glyceraldehyde-3-phosphate dehydrogenase	36	9	51	47.435
P04083	Annexin A1	38.7	9	29	34.03
P07195	L-lactate dehydrogenase B chain	36.6	11	43	31.115
P12236	ADP/ATP translocase 3	32.8	11	43	27.395
P05141	ADP/ATP translocase 2	32.8	11	35	23.555
P60174	Triosephosphate isomerase	30.8	7	38	22.9
E7EX29	14-3-3 protein zeta/delta (Fragment)	28	5	27	22.402
P23396	40S ribosomal protein S3	26.7	7	40	21.797
P00338	L-lactate dehydrogenase A chain	36.7	11	44	20.632
A0A286YEY5	Immunoglobulin heavy constant alpha 2 (Fragment)	42.3	5	26	20.53
A0A286YEY1	Immunoglobulin heavy constant alpha 1 (Fragment)	42.8	6	24	20.358
P12273	Prolactin-inducible protein	16.6	6	57	20.31
O60218	Aldo-keto reductase family 1 member B10	36	7	34	20.071
P15880	40S ribosomal protein S2	31.3	7	30	19.744
P63244	Receptor of activated protein C kinase 1	35.1	8	29	19.062
P62753	40S ribosomal protein S6	28.7	6	30	18.73
Q07021	Complement component 1 Q subcomponent-binding protein, mitochondrial	31.3	4	27	18.635
P15121	Aldose reductase	35.8	7	34	18.634
P09525	Annexin A4	35.9	6	25	17.655
Q16698	2,4-dienoyl-CoA reductase, mitochondrial	36	6	30	17.179
P13804	Electron transfer flavoprotein subunit alpha, mitochondrial	35.1	5	22	16.658
P27348	14-3-3 protein theta	27.7	6	30	16.143
P09651	Heterogeneous nuclear ribonucleoprotein A1	38.7	5	20	16.081
Q96DA0	Zymogen granule protein 16 homolog B	22.7	3	23	16.061
P62701	40S ribosomal protein S4, X isoform	29.6	7	26	14.299

P08758	Annexin A5	35.9	6	22	14.295
P61981	14-3-3 protein gamma	28.3	5	21	13.631
P18669	Phosphoglycerate mutase 1	28.8	4	31	13.521
P40926	Malate dehydrogenase, mitochondrial OS=Homo sapiens OX=9606 GN=MDH2 PE=1 SV=3	35.5	5	16	11.787
P31946	14-3-3 protein beta/alpha	28.1	4	20	11.002
P62258	14-3-3 protein epsilon	29.2	4	18	10.967
P18124	60S ribosomal protein L7	29.2	5	17	10.388
E9PB61	THO complex subunit 4	27.5	3	26	9.747
P62424	60S ribosomal protein L7a	30	4	19	9.609
P01833	Polymeric immunoglobulin receptor	83.2	4	6	8.763
P68104	Elongation factor 1-alpha 1	50.1	3	9	8.537
P01834	Immunoglobulin kappa constant	11.8	3	49	7.959
P06493	Cyclin-dependent kinase 1	34.1	5	21	7.718
P25786	Proteasome subunit alpha type-1	29.5	4	18	7.698
Q9Y676	28S ribosomal protein S18b, mitochondrial	29.4	3	18	7.224
P0DOY2	Immunoglobulin lambda constant 2	11.3	2	24	6.904
P15559	NAD(P)H dehydrogenase [quinone] 1	30.8	4	18	6.822
P60709	Actin, cytoplasmic 1	41.7	5	16	6.718
Q13011	Delta(3,5)-Delta(2,4)-dienoyl-CoA isomerase, mitochondrial	35.8	2	9	6.663
E9PAV3	Nascent polypeptide-associated complex subunit alpha, muscle-specific form	205.3	2	1	6.434
P62906	60S ribosomal protein L10a	24.8	1	6	6.425
P31947	14-3-3 protein sigma	27.8	3	12	6.382
Q04917	14-3-3 protein eta	28.2	3	13	6.341
F5H5D3	Tubulin alpha chain	57.7	2	6	6.333
O14818	Proteasome subunit alpha type-7	27.9	3	16	6.016
P12429	Annexin A3	36.4	3	11	5.932
Q15717	ELAV-like protein 1	36.1	2	9	5.863
P46777	60S ribosomal protein L5	34.3	2	11	5.676
Q96AG4	Leucine-rich repeat-containing protein 59	34.9	3	10	5.298
Q00325	Phosphate carrier protein, mitochondrial	40.1	2	6	5.226
P04745	Alpha-amylase 1	57.7	3	7	5.206
Q9UIJ7	GTP:AMP phosphotransferase AK3, mitochondrial	25.6	1	6	5.078
Q8WXX5	DnaJ homolog subfamily C member 9	29.9	2	9	5.062

P00491	Purine nucleoside phosphorylase	32.1	2	14	4.903
Q9H9B4	Sideroflexin-1	35.6	3	11	4.581
P61247	40S ribosomal protein S3a	29.9	3	13	4.406
Q14847	LIM and SH3 domain protein 1	29.7	3	14	4.345
B1AK88	Capping protein (Actin filament) muscle Z-line, beta, isoform CRA_d	33.8	3	8	4.248
O75828	Carbonyl reductase [NADPH] 3	30.8	2	12	4.246
P35270	Sepiapterin reductase	28	1	7	4.138
Q13151	Heterogeneous nuclear ribonucleoprotein A0	30.8	1	5	3.991
Q13084	39S ribosomal protein L28, mitochondrial	30.1	1	9	3.838
P62917	60S ribosomal protein L8	28	2	11	3.819
P61626	Lysozyme C	16.5	2	14	3.8
Q13162	Peroxiredoxin-4	30.5	2	9	3.701
P16152	Carbonyl reductase [NADPH] 1	30.4	2	12	3.687
P30040	Endoplasmic reticulum resident protein 29	29	2	10	3.642
Q06323	Proteasome activator complex subunit 1	28.7	1	5	3.625
P26373	60S ribosomal protein L13	24.2	2	9	3.57
Q5HYB6	Epididymis luminal protein 189	27.2	2	11	3.499
P56537	Eukaryotic translation initiation factor 6	26.6	1	10	3.47
A0A087X 1Z3	Proteasome activator complex subunit 2	29.1	1	5	3.432
P01591	Immunoglobulin J chain	18.1	1	8	3.296
P62873	Guanine nucleotide-binding protein G(I)/G(S)/G(T) subunit beta-1	37.4	1	3	3.083
P81605	Dermcidin	11.3	2	23	3.06
P05388	60S acidic ribosomal protein P0	34.3	1	4	3.057
P36542	ATP synthase subunit gamma, mitochondrial	33	2	7	3.042
P84098	60S ribosomal protein L19	23.5	1	9	2.943
P09601	Heme oxygenase 1	32.8	1	8	2.942
P30041	Peroxiredoxin-6	25	2	9	2.897
P22626	Heterogeneous nuclear ribonucleoproteins A2/B1	37.4	1	3	2.696
P52895	Aldo-keto reductase family 1 member C2	36.7	1	7	2.619
Q15366	Poly(rC)-binding protein 2	38.6	2	6	2.598
P13928	Annexin A8	36.9	1	5	2.59
Q14165	Malectin	32.2	1	4	2.577
P62241	40S ribosomal protein S8	24.2	1	5	2.479

O00299	Chloride intracellular channel protein 1	26.9	2	9	2.322
O15144	Actin-related protein 2/3 complex subunit 2	34.3	1	4	2.269
Q02978	Mitochondrial 2-oxoglutarate/malate carrier protein	34	2	5	2.241
Q9BRL6	Serine/arginine-rich splicing factor 8	32.3	1	3	2.223
Q9NZT1	Calmodulin-like protein 5	15.9	1	16	2.217
P25789	Proteasome subunit alpha type-4	29.5	1	4	2.157
J3QQ67	60S ribosomal protein L18 (Fragment)	21.8	1	7	2.075
Q9HC84	Mucin-5B	596	1	1	2.044
Q9NPF4	Probable tRNA N6-adenosine threonylcarbamoyltransferase	36.4	1	5	2.017
P25311	Zinc-alpha-2-glycoprotein	34.2	1	4	1.9
Q96HS1	Serine/threonine-protein phosphatase PGAM5, mitochondrial	32	1	3	1.896
P21796	Voltage-dependent anion-selective channel protein 1	30.8	1	4	1.887
A0A087WYR3	Tumor protein D54	23.8	1	9	1.886
P25705	ATP synthase subunit alpha, mitochondrial	59.7	1	2	1.874
P35030	Trypsin-3	32.5	1	4	1.834
F8W0W8	Serine/threonine-protein phosphatase	38.2	1	5	1.795
G5EA09	Syndecan binding protein (Syntenin), isoform CRA_a	34.8	1	3	1.651
P24534	Elongation factor 1-beta	24.7	1	6	1.639
Q9Y399	28S ribosomal protein S2, mitochondrial	33.2	1	3	1.592
P48556	26S proteasome non-ATPase regulatory subunit 8	39.6	1	2	1.574
G3V5Z7	Proteasome subunit alpha type	28.1	1	5	1.574
P38646	Stress-70 protein, mitochondrial	73.6	1	2	1.529
Q16629	Serine/arginine-rich splicing factor 7	27.4	1	5	1.523
Q9H3N1	Thioredoxin-related transmembrane protein 1	31.8	1	3	1.511

Whole A549 cell lysates were prepared for immunoprecipitation using an anti-PHB2 antibody, and immunocomplexes were analyzed using LC-MS/MS.

Table S3. Control IgG interacting partners were identified with LC-MS/MS.

Accession	Description	MW [kDa]	Peptides (n)	Coverage [%]	Sum PEP Score
P04264	Keratin, type II cytoskeletal 1	66	48	68	134.438
P68133	Actin, alpha skeletal muscle	42	8	30	16.672
P16615	Sarcoplasmic/endoplasmic reticulum calcium ATPase 2	114.7	3	5	11.009
Q9UKX2	Myosin-2	222.9	7	5	8.81
P60709	Actin, cytoplasmic 1	41.7	5	16	8.573
Q9Y623	Myosin-4	222.9	6	4	8.152
P25705	ATP synthase subunit alpha, mitochondrial	59.7	2	6	7.593
H3BPK4	Myosin regulatory light chain 2, skeletal muscle isoform (Fragment)	22	4	29	7.075
Q5T749	Keratinocyte proline-rich protein	64.1	6	15	6.97
F5H5D3	Tubulin alpha chain	57.7	3	8	5.75
A0A0A0 MTS7	Titin	3992.2	1	0	4.808
P06733	Alpha-enolase	47.1	1	5	4.503
P14923	Junction plakoglobin	81.7	3	6	3.905
P06732	Creatine kinase M-type	43.1	2	7	3.785
Q9UKX3	Myosin-13	223.5	2	1	3.348
P68371	Tubulin beta-4B chain	49.8	2	6	3.137
Q6ZN40	Tropomyosin 1 (Alpha), isoform CRA_f	37.4	2	6	3.025
P68104	Elongation factor 1-alpha 1	50.1	3	7	2.809
Q02413	Desmoglein-1	113.7	2	3	2.801
P45378	Troponin T, fast skeletal muscle	31.8	1	6	2.548
A0A087 WSZ2	Alpha-actinin-3	107.6	1	1	2.486
P62805	Histone H4	11.4	2	21	2.396
P11217	Glycogen phosphorylase, muscle form	97	1	2	2.355
A0A286Y ES1	Immunoglobulin heavy constant gamma 3 (Fragment)	49.1	2	4	2.203
P06576	ATP synthase subunit beta, mitochondrial	56.5	2	6	2.187
P05976	Myosin light chain 1/3, skeletal muscle isoform	21.1	2	9	1.888
Q08554	Desmocollin-1	99.9	2	3	1.667

Whole A549 cell lysates were prepared for immunoprecipitation using a control IgG antibody, and immunocomplexes were analyzed using LC-MS/MS.
